# Multi-trait selection for mean performance and stability of maize hybrids in mega-environments delineated using envirotyping techniques

**DOI:** 10.3389/fpls.2022.1030521

**Published:** 2022-11-14

**Authors:** Haiwang Yue, Tiago Olivoto, Junzhou Bu, Jie Li, Jianwei Wei, Junliang Xie, Shuping Chen, Haicheng Peng, Maicon Nardino, Xuwen Jiang

**Affiliations:** ^1^ Hebei Provincial Key Laboratory of Crops Drought Resistance Research, Dryland Farming Institute, Hebei Academy of Agriculture and Forestry Sciences, Hengshui, China; ^2^ Department of Plant Science, Center of Agrarian Sciences, Federal University of Santa Catarina, Florianópolis, SC, Brazil; ^3^ Department of Agronomy, Federal University of Viçosa, Viçosa, MG, Brazil; ^4^ Maize Research Institute, Qingdao Agricultural University, Qingdao, China

**Keywords:** maize hybrid, mega-environment delineation, genotype-environment interaction, climatic variables, MTMPS

## Abstract

Under global climate changes, understanding climate variables that are most associated with environmental kinships can contribute to improving the success of hybrid selection, mainly in environments with high climate variations. The main goal of this study is to integrate envirotyping techniques and multi-trait selection for mean performance and the stability of maize genotypes growing in the Huanghuaihai plain in China. A panel of 26 maize hybrids growing in 10 locations in two crop seasons was evaluated for 9 traits. Considering 20 years of climate information and 19 environmental covariables, we identified four mega-environments (ME) in the Huanghuaihai plain which grouped locations that share similar long-term weather patterns. All the studied traits were significantly affected by the genotype × mega-environment × year interaction, suggesting that evaluating maize stability using single-year, multi-environment trials may provide misleading recommendations. Counterintuitively, the highest yields were not observed in the locations with higher accumulated rainfall, leading to the hypothesis that lower vapor pressure deficit, minimum temperatures, and high relative humidity are climate variables that –under no water restriction– reduce plant transpiration and consequently the yield. Utilizing the multi-trait mean performance and stability index (MTMPS) prominent hybrids with satisfactory mean performance and stability across cultivation years were identified. G23 and G25 were selected within three out of the four mega-environments, being considered the most stable and widely adapted hybrids from the panel. The G5 showed satisfactory yield and stability across contrasting years in the drier, warmer, and with higher vapor pressure deficit mega-environment, which included locations in the Hubei province. Overall, this study opens the door to a more systematic and dynamic characterization of the environment to better understand the genotype-by-environment interaction in multi-environment trials.

## 1 Introduction

Maize *(Zea mays* L.) is an annual herb belonging to the grass family *Poaceae* in botanical classification. With its high-yielding, diverse uses, and wide adaptability, maize has surpassed rice (*Oryza sativa* L.) and wheat (*Triticum aestivum* L.) as the most important cereal crop in the world (Haarhoff and Swanepoel, 2018). If the world population grows to 10 billion, it will need 70% more food than can be accomplished today (Hickey et al., 2019). Maize is estimated to account for more than half of future cereal demand growth. Thus, there is a huge stream of innovation for maize breeders when trying to significantly increase maize productivity in an environmentally sensitive way (Yan and Tan, 2019). Since 2013, maize has become the largest crop in China in terms of planting area and production. China’s maize planting area has exceeded 37 million hectares, with a total production of more than 215 million tons, accounting for one-quarter and one-fifth of the world’s maize area and production, respectively (Hou et al., 2020).

**Figure 1:**
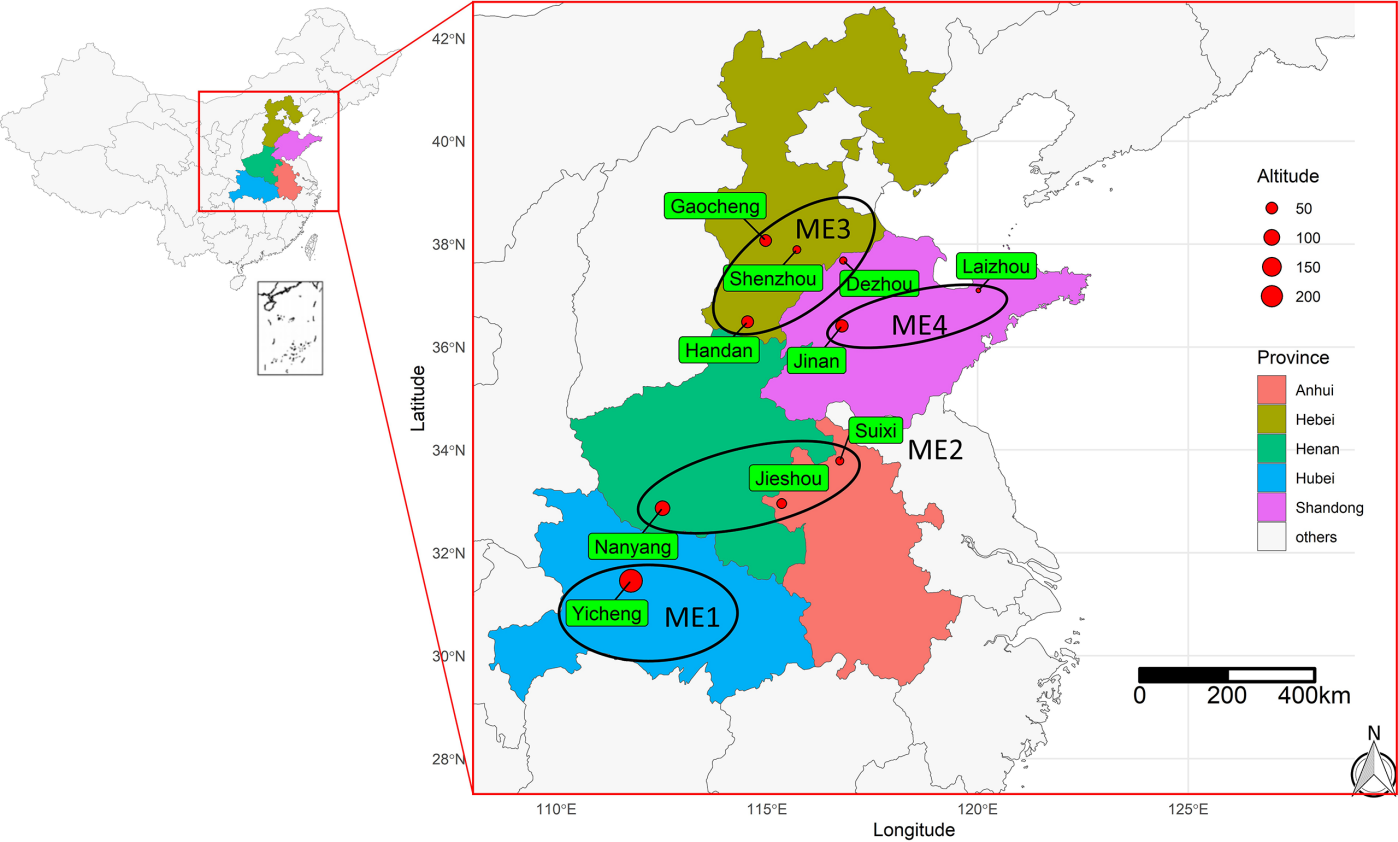
Geographical information of the 10 test locations for the trials conducted during 2019 and 2020. The ellipses show the four delineated mega-environments (ME) based on long-term (20 years) climate information.

Maize production is divided into spring maize, summer maize, and autumn maize according to the growth period in China. The Huanghuaihai (HHH) plain (**Figure 1**) is the largest concentrated summer maize planting area in China, accounting for 31.86% and 30.68% of the country’s total area and yield, respectively (Zhai et al., 2022). The meteorological conditions in the HHH plain are complex, often encountering high temperatures, heat damage, cloudy rain and lack of sunshine, and the invasion of various diseases, which make maize yields vary greatly from year to year (Wang et al., 2020; Shi et al., 2021; Yue et al., 2022b). Unencouraging climate change projections suggest that the temperature increase might be a key factor affecting the drought risk in HHH (Yue et al., 2022c). This may put at risk the breeding efforts that generated maize hybrids for this area and increase the challenges of breeding programs that aim to release new hybrids (Rizzo et al., 2022). Therefore, there is an urgent need to better understand the genotype-by-environment interaction (GEI) in this region to develop and improve climate-resilient maize hybrids that are thoroughly evaluated in different locations and years/seasons before release. This can be one of the most effective ways for increasing maize production in HHH under new challenges from climate change. In this context, identifying climate-related variables that are most associated with the variations of hybrids within environments is crucial for defining management and/or selection strategies for breeding new summer maize hybrids in the HHH plain region (Yue et al., 2021).

Although the challenge of developing abiotic stress-tolerant maize hybrids has generated a large literature, most practical breeding efforts have also focused on breeding for genetic variation, heritability for grain yield progress under favorable conditions (Bänziger et al., 2006; Fischer and Edmeades, 2010). Grain yield and its components are very complex agronomic traits influenced by genotype (G), environment (E), and their interactions (GEI). The GEI makes the genotype-to-phenotype relationship environment-dependent, which makes the selection of widely adapted hybrids more difficult (Ebdon and Gauch, 2002) and occurs due to the differential response of a given genotype to a given environment stimulated by both biotic, abiotic, or an interaction between them (Nardino et al., 2022). In maize, for example, high temperatures (> 35°C) during flowering generate a cascade effect that starts with the reduction of RuBPCase activity by downregulating genes Zm0001d052595 and Zm0001d004894 which limited photosynthesis and consequently affects maize growth and development (Niu et al., 2021). As a consequence, maize grain yield (GY) is reduced mainly by reducing kernel number per ear, a process associated with carbohydrate metabolic disorders, where a lower carbohydrate availability leads to kernel abortion under post-pollination heat stress conditions (Dong et al., 2021; Niu et al., 2021). Therefore, even if the two environments are strictly similar (e.g., in terms of soil fertility, average temperatures, and rainfall precipitation), extreme events can affect the plants differently, mainly depending on the crop stage they occur.

The correct interpretation of GEI effects in multi-environment trials (METs) can help to select genotypes with high-yielding and stable under different environmental conditions, and even select special genotypes for a certain environment (Vaezi et al., 2019; Alizadeh et al., 2022). During breeding practice, breeders often measure many traits related to yield and are faced with the problem of selecting stable and superior genotypes based on multiple traits. The multi-trait stability index (MTSI) has been successfully used for selecting superior genotypes based on multiple traits (Koundinya et al., 2021; Singamsetti et al., 2021; Farhad et al., 2022; Lima et al., 2022; Padmaja et al., 2022), and has a tremendous potential to combine morpho-physiological and yield traits aiming at selecting hybrids under optimal and stress conditions (Balbaa et al., 2022).

Identifying hybrids that rise to the top in terms of multiple criteria from a set of evaluation sites is important but does not contribute significantly to new insights into maize evaluation research. Choosing an ideal genotype (stable across all environments) may ignore specific adaptations, mainly under the climate changes in view (Lopes et al., 2015). Therefore, identifying mega-environments that include locations that share similar long-term weather patterns can be an alternative to better explore the GE interaction in favor of better selection gains, mainly in a region/environment with high variations among the locations/seasons (Costa-Neto et al., 2021a).

In this sense, the main goal of this study is to use envirotyping techniques to delineate mega-environments across the Huanghuaihai plain in China, and to select superior hybrids within each mega-environment that are stable across the cultivation years based on multi-trait. Overall, this study provides new insights into a more systematic and dynamic characterization of the test environments, helping breeders to make better strategic decisions toward an effective multivariate selection in maize breeding programs.

## 2 Materials and methods

### 2.1 Plant materials, locations, and experimental design

The experimental material consisted of 26 maize genotypes including one local check hybrid, Zhengdan 958 (**Table 1**). This study was carried out in ten environments (**Figure 1**) across five provinces ranging from middle temperate zone to the warm temperate zone, at an elevation from 18 to 235 m above mean sea level spreading across the states of Hebei, Shandong, Anhui, Henan, and Hubei during 2019-2020. The field experiment used a randomized complete block design with three replicates. The seeds of each tested genotype were provided by Dryland Farming Institute, Hebei Academy of Agriculture and Forestry Sciences, and healthy and coating seeds were selected for this study before sowing. The plot at each location was composed of 5 rows with 0.6 m spacing between rows, and the area of each plot had 20.1 m^2^ in size. The planting density of each genotype was strictly controlled at 7.5 plants m^-2^, and the field management applied during the experiment was similar to the management practiced by farmers.

**Table 1:** Basic information of the 26 tested maize hybrids.

Code	Genotype	Parentage	Plant height (cm)	Ear height (cm)	Origin	Maturity	Input requirements
G1	Xianyu335	PH6WC×PH4CV	286	103	Liaoning	Medium	High
G2	Hengyu1702	H1027×HC705	255	98	Hebei	Medium	Medium
G3	Hengyu7182	H103×H102	245	87	Hebei	Early	Low
G4	Jiuheng517	H103×H92	243	78	Hebei	Early	Low
G5	Huanong138	B105×J66	281	102	Beijing	Medium	High
G6	Hengyu1587	H58×H59	254	101	Hebei	Medium	Medium
G7	Denongli988	Wan73-1×M518	280	120	Shandong	Late	High
G8	Xundan29	X313×X66	258	117	Henan	Medium	High
G9	Hengyu7188	HB4×H88	260	97	Hebei	Medium	Low
G10	Hengyu321	H14×H13	275	115	Hebei	Medium	Medium
G11	Hengyu1182	H11×H82	268	109	Hebei	Early	Low
G12	Heng110	H58/H59	242	82	Hebei	Early	Low
G13	Liyu16	953×L91158	264	123	Hebei	Late	High
G14	Denghai662	DH371×DH382	272	98	Shandong	Late	Medium
G15	Heng9	H1027×H765	244	79	Hebei	Early	Medium
G16	Zhengjie1	L112×Lx9801	259	92	Shandong	Medium	High
G17	Nongle988	NL278×NL167	250	113	Henan	Late	High
G18	Lianchuang5	CT07×Lx9801	270	106	Henan	Early	High
G19	Tunyu808	T88×T172	253	110	Tianjin	Medium	High
G20	Zhengdan958	Z58×C7-2	250	110	Henan	Late	Low
G21	Meiyu5	758×HC7	255	107	Henan	Early	Medium
G22	Lile66	C28×CH05	270	108	Henan	Late	High
G23	Liyu86	L5895×L5012	267	114	Hebei	Medium	High
G24	Hengdan6272	H462×H72	261	126	Hebei	Medium	Medium
G25	Weike702	WK858×WK798-2	252	107	Henan	Late	High
G26	Shengrui999	S68×S62	250	107	Henan	Medium	Medium

### 2.2 Morphological data recording

A total of 9 yield-related agronomic traits were recorded in this study. Agronomic traits viz., grain yield (GY, t ha^-1^) was manually harvested from the middle three rows, adjusting the moisture to 14% and converting the unit to tons per hectare; grain moisture content (GMC, %), measured from each plant at each plot; plant height (PH, cm), measured from the base of the root to the top of the tassel; ear height (EH, cm), measured from the base of the root to the stalk of the ear; ear length (EL, cm), measured from the line up 10 ears, and dividing the data obtained by 10; ear row (ER), counting the total number of rows in each ear; bare tip length (BTL, cm), measured from the top part with no grains (if any) to the part with grains; grain weight per ear (GWE, g) and 100-seed weight (HSW, g) (Yue et al., 2022a).

### 2.3 Statistical Analysis

#### 2.3.1 Mega-environment delineation

Aiming at defining mega-environments with a similar long-term pattern of climate characteristics, we used the function get_wheater() function from the R package EnvRtype (Costa-Neto et al., 2021b) to download a 20-year (2001-2020), daily-basis weather data for 19 environmental covariables (EC) (**Table 2**). For each year, we considered the period between May and October, which cover the maize growing season in the studied locations. EnvRtype is a very practical package that downloads and processes remote weather data from “NASA’s Prediction of Worldwide Energy Resources” (NASA/POWER, https://power.larc.nasa.gov/). Experimental results show that NASA/POWER can be used as a source of climatic data for agricultural activities with reasonable confidence for regional and national spatial scales (Monteiro et al., 2018). A correlation analysis between NASA/POWER data and observed data at Shenzhou location (**Supplementary Figure S1**) showed a high concordance for temperature variables and sunshine duration (*r* > 0.91, *P*< 0.01), and relative humidity (*r* = 0.88, *P*< 0.01). For rainfall precipitation, a lower agreement (*r* > 0.54, P< 0.01) was observed. For the accumulated rainfall precipitation, NASA/POWER tended to overestimate the real observed precipitation.

**Table 2:** List of environmental covariables used in the study.

Source	Environmental factor	Unit
Nasa POWER^a^	Insolation Incident on a Horizontal Surface	MJ m^−2^ day^−1^
	Downward Thermal Infrared (Longwave) Radiative Flux	MJ m^−2^ day^−1^
	Extraterrestrial radiation	MJ m^−2^ day^−1^
	Wind speed at 2 m above the surface of the earth	m s^−1^
	Minimum air temperature at 2 above the surface of the earth	°C day ^−1^
	Average air temperature at 2 above the surface of the earth	°C day ^−1^
	Maximum air temperature at 2 above the surface of the earth	°C day ^−1^
	Dew-point temperature at 2 m above the surface of the earth	°C day ^−1^
	Relative air humidity at 2 above the surface of the earth	%
	Rainfall precipitation	mm day ^−1^
Calculated^b^	Temperature range	°C d^−1^
	Potential Evapotranspiration	mm d^−1^
	Deficit by precipitation	mm d^−1^
	Vapor Pressure Deficit	kPa d^−1^
	Slope of saturation vapor pressure curve	Kpa °C d^−1^
	Effect of temperature on radiation-use efficiency	–
	Growing Degree Day	°C day^−1^
	Actual duration of sunshine	hour
	Daylight hours	hour

^a^Estimated from NASA orbital sensors (Sparks, 2018); ^b^ processed using concepts from Allen et al. (1998) and Soltani and Sinclair (2012).

The 19 EC observed in each location were used to create the called envirotype covariable matrix W that was further used to compute environmental kinships using the function W_matrix() of the EnvRtype package (Costa-Neto et al., 2021b) as proposed by (Costa-Neto et al., 2021a). To better capture the temporal variation of the environmental information across months of the year, six monthly periods were considered (May-October). Therefore, each one of the 2280 variables (20 years × 19 variables × 6 periods = 2280) has become an envirotype descriptor of environmental relatedness. Finally, quality control was done by removing covariables that exceeded ±3SD, where SD is the standard deviation of the covariables across environments (Costa-Neto et al., 2021a). Then, using the W (10 rows ×  2280 columns) matrix, we calculated an enviromic kernel (equivalent to a genomic relationship), using the function env kernel() of the EnvRtype package (Costa-Neto et al., 2021b), as follows:


$$K_{E}=\;\frac{WW'}{trace\left(WW^{\prime }\right)/nrow\left(W\right)}$$

where *K*_*E*_  is the enviromic-based kernel for the similarity between environments and *W* is the matrix of envirotype descriptors. To identify mega-environments, a hierarchical clustering (average method) was applied to *K*_*E*_ .

Finally, to visually understand the relationships between environmental variables and their association with the location study, we conducted a Principal Component Analysis (PCA) with a two-way table with the average values for the environmental variables (columns) for each location (rows). A biplot was produced with the function fviz_pca_biplot() from the R package factoextra (Kassambara and Mundt, 2020).

#### 2.3.2 Environmental typology of the trials

To characterize the climate data observed during the experimental period, we used the function env_typing() of the R package EnvRtype to create environmental typologies based on quantilic limits of the 19 EC (**Table 2**) collected between the sowing and harvesting of each trial. To better capture the temporal variation of the environmental information across crop development, the crop cycles were divided into five main phenological stages in days after sowing (DAS): 0-14 (Initial growing); 15-35 DAS (leaf expansion I, V4-V8); 36-65 DAS (leaf expansion II, V8 - VT); 66-90 DAS (flowering); and 91-120 (grain filling). For each YEAR-ME-stage combination, frequency distributions were computed considering the quantiles 0.01, 0.25, 0.50, 0.75, 0.975, and 0.99; with this, extreme values (e.g., high temperatures) can be identified.

#### 2.3.3 Variance component analysis

To estimate the effect of the respective influences of ME and year on the genotype behavior, for each trait we fitted a linear random-effects model (only intercept as fixed) was fitted using the lmer() function from the lme4 R package (Bates et al., 2015), according to the following model:


$$y_{ijkn}=\mu +G_{i}+M_{j}+Y_{k}+GM_{ij}+GY_{ik}+MY_{jk}+GMY_{ijk}+REP_{n\left(j:k\right)}+\epsilon_{ijkn}$$

where *y*_*ijkn*_ is the trait scores of *i-*th genotype observed in the *n-*th replicate, which is nested within the *j*-th mega-environment of the *k-*th year; *μ*  is the grand mean; *G*_*i*_ , *M*_*j*_ , and *Y*_*k*_ are the main effects of genotype, mega-environment, and year; *GM*_*ij*_ is the interaction effect of genotype and mega-environment; *GY*_*ik*_ is the interaction effect of genotype and year; *MY*_*jk*_ is the interaction effect of mega-environment and year; *GMY*_*ijk*_ is the interaction of genotype, mega-environment, and year; *REP*_*n*(*j*:*k*)_ is the effect of the replicate *n* (assumed to be the combination of location and blocks) nested within the mega-environment and year; and *ϵ*_*ijkn*_ is the random error associated to *y*_*ijkn*_ . Variance components and genetic parameters were estimated using Restricted Maximum Likelihood, REML (Dempster et al., 1977). Significance testing for random effects was done by the likelihood ratio test (LRT) comparing a complete model (with all terms) and a model without the term under test. The broad-sense heritability on a genotype-mean basis (H^2^) was computed as the ratio between genotypic variance ( ${\text{σ}}_{G}^{2})$ and variance of a genotype mean ( ${\text{σ}}_{P}^{2})$ , as follows (Yan, 2014; Schmidt et al., 2019).


$${\text{H}}^{2}=\frac{{\text{σ}}_{G}^{2}}{{\text{σ}}_{P}^{2}}=\frac{{\text{σ}}_{G}^{2}}{{\text{σ}}_{G}^{2}+\frac{{\text{σ}}_{GY}^{2}}{K}+\frac{{\text{σ}}_{GM}^{2}}{J}+\frac{{\text{σ}}_{GMY}^{2}}{JK}+\frac{{\text{σ}}_{\epsilon }^{2}}{\sum_{k=1}^{K}N}}$$

Where J, K, and N are the numbers of mega-environments, years, and combinations of location/blocks, respectively. ${\text{σ}}_{G}^{2},\;{\text{σ}}_{GY}^{2},\;{\text{σ}}_{GM}^{2},{\text{and σ}}_{GMY}^{2}$ are the variances of GEN, GEN×YEAR interaction, GEN×ME interaction, and the GEN×YEAR×ME interaction, respectively; ${\text{σ}}_{\epsilon }^{2}$ is the residual variance. An H^2^ close to 1 means that any observed differences among the genotypic effects are completely due to genetic differences; On the other hand, an H^2^ close to 0 means that observed genotypic differences, are due to either genotype-by-environment interactions or experimental errors (Yan, 2014). Finally, we compute the accuracy (Ac) as follows:


$$Ac\;=\sqrt{{\text{H}}^{2}}$$

Both the percentage of the variance of phenotypic mean values (considering each term of the random-effect model) and the percentage of the variance of a genotype mean (contribution of each component to the ${\text{σ}}_{P}^{2}$ ) were presented as filled bar plots.

#### 2.3.4 Mean performance and stability of single trait

Genotype selection was performed within each delineated ME aiming at selecting genotypes that combine desired performance within the ME and are stable across years; such a genotype would be desired by both farmers and breeders. First, for each ME, the average performance of the *I* genotypes in the *K* years ( ${\bar{Y}}_{ik})$ was computed. Then, the Wricke’s Ecovalence (*W*_*i*_ ) was used as a measure of the genotypic stability across the years and was computed as follows:


$$W_{i}=\sum_{k=1}^{K}\left({\bar{Y}}_{ik}-{\bar{Y}}_{i.}-{\bar{Y}}_{.k}+{\bar{Y}}_{..}\right)$$

Genotypes with low values of *W*_*i*_ have smaller deviations from the mean across years being then more stable. To account for both mean performance and stability (MPS*_i_*) of genotypes, we adapted the concept of the WAASBY index, which is based on the weighted average of absolute scores from the singular value decomposition of the matrix of best linear unbiased prediction (BLUP) for the GEI effects generated by a linear mixed-effect model (LMM) and response variable (Olivoto et al., 2019a), by replacing the weighted average of absolute scores (WAASB) with *W*_*i*_ as stability measure, since to compute WAASB at least two Interaction Principal Component Axes are needed. The MPS*_i_* was computed as follows:


$$MPS_{i}=\frac{\left(rY_{i}\times \theta_{Y}\right)+\left(rW_{i}\times \theta_{s}\right)}{\theta_{Y}+\theta_{s}}$$

where *MPS_i_* is the superiority index for genotype *i* that weights between mean performance and stability; *θ*_*Y*_ and *θ*_*s*_ are the weights for mean performance and stability, respectively; *rY*_*i*_ and *rW*_*i*_ are the rescaled values for mean performance $\bar{Y_{i}}$ and stability (*W*_*i*_) , respectively of the genotype *i*. Here, we used *θ*_*Y*_=70 and *θ*_*s*_=30 to account for a higher weight for mean performance, since selecting highly stable hybrids that do not perform well is not desired. The rescaled values were computed as follows:


$$rY_{i}=rW_{i}=\frac{nma-nmi}{oma-omi}\times \left(o_{i}-oma\right)+nma$$

where *nma* and *nmi* are the new maximum and minimum values after rescaling; *oma* and *omi* are the original maximum and minimum value, and o*_i_* is the original value for the response variable (or ecovalence value) for the genotype *i*. For *W*_*i*_ and the traits GMC, PH, EH, and BTL in which lower values are desired, we used *nma* = 0 and *nmi* = 100. So, the genotype with the lowest mean and lowest *W*_*i*_ would have *rYi =*100 and *rWi =*100 after rescaling. For, GY, EL, ER, GWE, and HSW in which higher values are desired, we used *nma* = 100 and *nmi* = 0. After rescaling all the traits, a two-way table *r* **M** *_qp_* with *q* rows (genotypes) and *p* columns (traits) was created. In *r* **M** *_qp_*, each column has a 0–100 range that considers the desired sense of selection (increase or decrease) and maintains the correlation structure of the original set of variables (Olivoto and Nardino, 2021). Additionally, to show how the ranking of genotypes is altered depending on the weight for mean performance and stability, for each ME we planned 21 scenarios changing the *θ*_*Y*_/*θ*_*s*_ ratio, as follows: 100/0, 95/5, 90/10,…, 0/100. To assist with intuitive interpretation, a heat map graph was produced. To compute these indexes we used the function mps() and wsmp() of the R package metan (Olivoto and Lúcio, 2020).

#### 2.3.5 Mean performance and stability of multiple traits

To account for the mean performance and stability of multiple traits, we used the function mgidi() of the metan R package to compute the multi-trait mean performance and stability index (MTMPS). The MTMPS is based on the concept of the Multi-trait stability index, MTSI (Olivoto et al., 2019b). The only difference between MTMPS and the MTSI is that in this study the MTMPS was computed considering the Wricke’s Ecovalence (*W*_*i*_ ) rather than the WAASB index. First, an exploratory factor analysis was computed with rM*_qp_* to group correlated variables into factors and compute the factorial scores for each genotype, as proposed by Olivoto and Nardino (2021):


X=μ+Lf+ϵ

where X is a *p*×1  vector of rescaled observations; μ is a *p*×1 vector of standardized means; L is a *p*×*f* matrix of factorial loadings; f is a *p*×1 vector of common factors; and ε is a *p*×1 vector of residuals, being *p* and *f* the number of traits and common factors retained, respectively. Initial loadings were obtained considering only factors with eigenvalues higher than one. After *varimax* rotation criteria (Kaiser, 1958) final loadings were obtained and were used to compute the genotype scores, as follows:


$$F=Z{\left(A^{T}R^{-1}\right)}^{T}$$

where F is a *q*×*f* matrix with the factorial scores; Z is a *q*×*p* matrix containing the standardized (zero mean and unit variance) r ***M*** *_qp_*; A is a *p*×*f* matrix of canonical loadings, and R is a *p*×*p* correlation matrix between the MPS values. *q* , *p* , and *f* represent the number of genotypes, traits, and retained factors, respectively.

Considering the rescaled values in r ***M*** *_qp_*, the ideotype would be the genotype that presents 100 for all analyzed traits; in other words, is the one that has the better performance and stability for all the analyzed traits. Thus, the ideotype was defined by a  (1×*p*)  vector ***I*** such that ***I*** =[100,100,…,100] . The genotype ranking was based on the Euclidean distance computed with the scores of each genotype to the score of the ideotype, as follows:


$$MTMPS_{i}={\left[\sum_{j=1}^{f}{\left(F_{ij}-F_{j}\right)}^{2}\right]}^{0.5}$$

Where *MTMPS*_*i*_ is the multi-trait mean performance index of the *i*th genotype, *F*_*ij*_ represents the *j*th scores of the *i*th genotype, *F*_*j*_  represents the *j*th scores of the ideotype. The genotypes with the lowest MTMPS values were closer to the ideotypes and thus showed high mean performance and better stability in the evaluated traits.

#### 2.3.6 Selection differentials

For each mega-environment, we assumed a selection intensity of ~23% (six selected hybrids). The selection differential in the percentage of population mean (Δ*S%*) was then computed for each trait as follows:


$$\text{Δ}S\%=(X_{s}-X_{o})/\;X_{o}\times 100$$

Where *X*_*s*_ and *X*_*o*_ are the mean phenotypic value of the selected genotypes and population mean, respectively.

#### 2.3.7 Statistical software

All statistical analyses in this study were performed using the R software 4.1.0 (R Core Team, 2022) with the packages and functions mentioned in each method.

## 3 Results

### 3.1 Environmental kinships and typology

#### 3.1.1 Historical data

**Figure 2:**
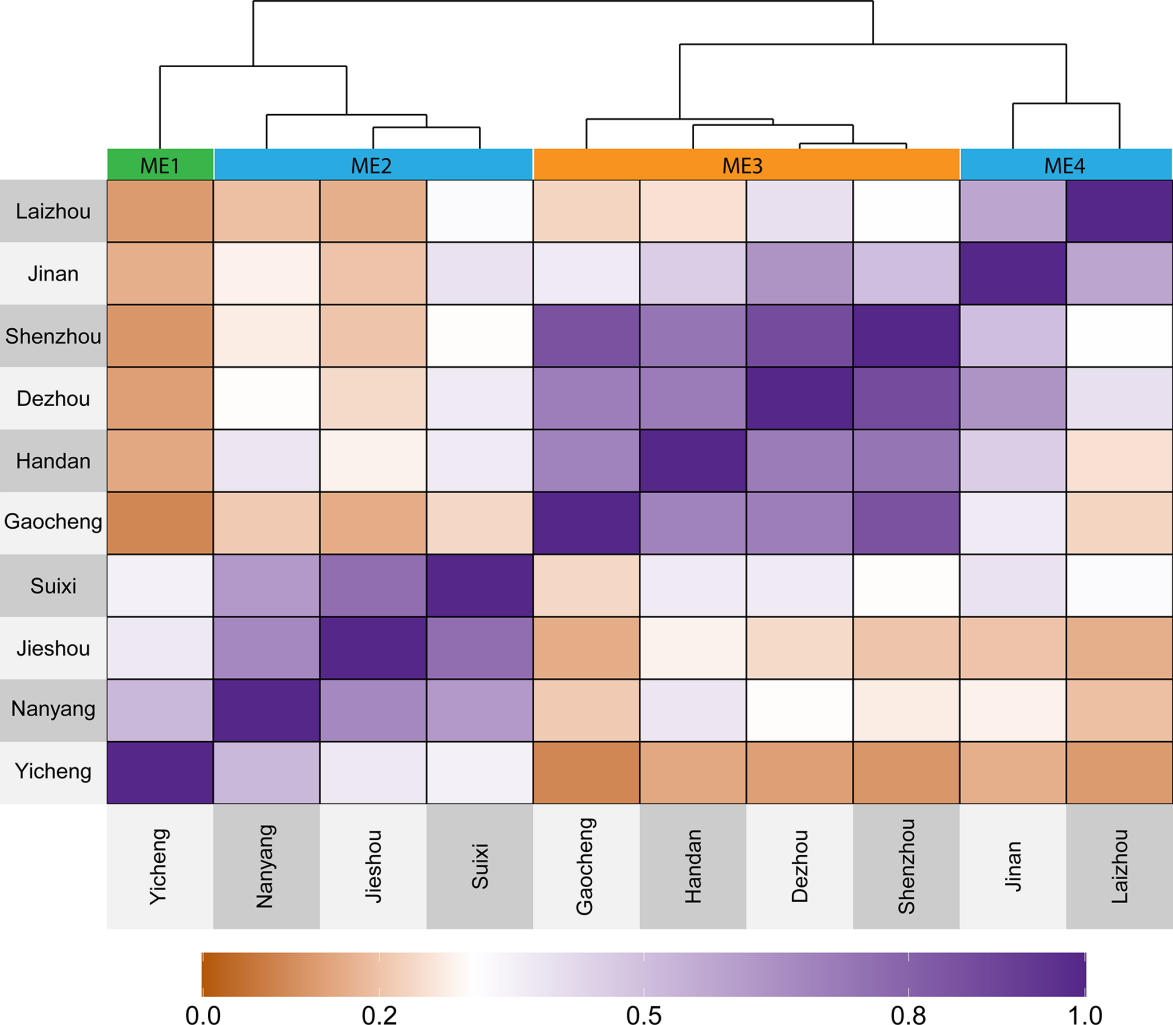
Heat map showing the delineated mega-environments considering the similarity based on 20 years of information for 19 environmental covariables.

Based on 20 years of climate information considering 19 environmental covariables, four mega-environments (ME) were delineated (**Figure 2**). The ME1 included only one location (Yicheng). The ME2 included Suixi, Jieshou, and Nanyang. The ME3 included Handan, Gaocheng, Shenzhou, and Dezhou; The ME4 included Jinan and Laizhou (**Figure 2**). The grouped ME were geographically close (**Figure 1**), suggesting that there is a relevant variation in the climate variables among the locations.

**Figure 3:**
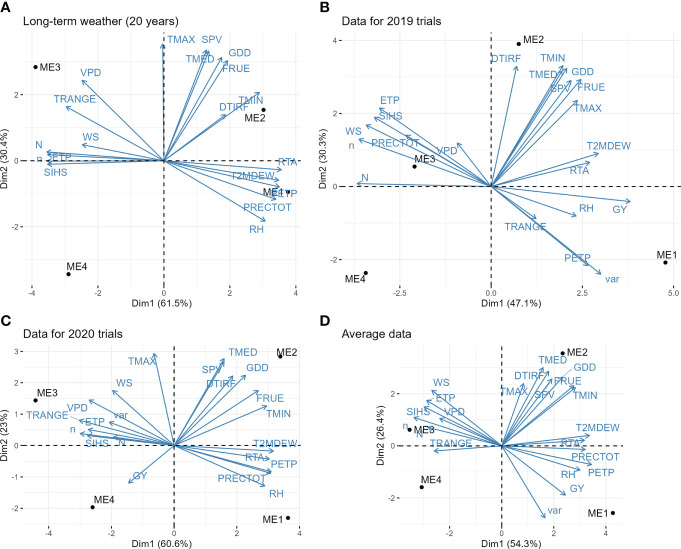
Biplot for the principal component analysis between environmental variables. **(A)** long-term pattern data (average of 20 years of climate information); **(B)** observed climate variables in the trials during 2019; **(C)** observed climate variables in the trials during 2020; **(D)** average information of the two years of trials. The variables are: grain yield (GY), genotype variance within mega-environment (var); average air temperature (TMED, °C d^-1^); minimum air temperature (TMIN, °C d^-1^); maximum air temperature (TMAX, °C d^-1^); dew-point temperature (T2MDEW, °C d^-1^) at 2 m above the surface of the earth at 2 m above the surface of the earth; total rainfall precipitation during the crop cycle (PRECTOT, mm); daily temperature range (TRANGE, °C d^-1^), deficit by precipitation (PETP, mm d^-1^); air relative humidity (RH, %), potential evapotranspiration (ETP, mm d^-1^); slope of saturation vapor pressure curve (SVP, Kpa °C d^-1^); vapor pressure deficit (VPD, kPa); Effect of temperature on radiation-use efficiency (FRUE); Growing Degree Day (GDD, °C day^−1^); Actual duration of sunshine (n, hours); Daylight hours (N, hours); Wind speed at 2 m above the surface of the earth (WS, m s^−1^); Extraterrestrial radiation (RTA, MJ m^−2^ day^-1^); Downward Thermal Infrared (Longwave) Radiative Flux (DTIRF, MJ m^−2^ day^-1^); Insolation Incident on a Horizontal Surface (SIHS, MJ m^−2^ day^−1^).

The extraterrestrial radiation (RTA), daylight hours (N), and deficit by precipitation (PETP) were the climate variables that most contributed to the environment scores (**Supplementary Figures S2-6**). The PCA biplot (**Figure 3A**) shows that ME1 is mainly characterized by having higher rainfall precipitation, relative humidity, and deficit by precipitation (higher deficit means more available water). The ME2 has the higher values for downward thermal infrared (Longwave) radiative flux. Contrary to ME1, ME3 has higher values for vapor pressure deficit and temperature range, meaning a drier environment (**Figure 3A**). The higher differences in the vapor pressure deficit of ME3 are specially observed from May to August (**Supplementary Figure S6**). The ME4 is mainly characterized by having a lower average temperature and consequently a small accumulation of growing degree days (**Figure 3A**).

The slope of the saturation vapor pressure curve, average temperature, and minimum and maximum temperature was positively associated. Temperature range and vapor pressure deficit were positively correlated but negatively correlated with relative humidity and precipitation, whereas potential evapotranspiration was strongly and positively correlated with extraterrestrial radiation (**Figure 3A**).

#### 3.1.2 Two years of trials

**Figure 3B** and **C** show the PCA biplot with the climate variables and MEs for the trials conducted in 2019 and 2020, respectively. It can be seen a high temporal (seasonal) effect, with different correlation patterns between the climate variables in the two years. For example, in 2019, rainfall precipitation and vapor pressure deficit were positively correlated whereas in 2020 were negatively correlated. This suggests that the interaction genotype x ME x year would have an important contribution to the phenotypic variance. In this case, identifying superior genotypes within ME that are stable across the years would be of great interest. Overall, ME1 had higher yields and rainfall precipitation. The higher temperatures were observed in ME2 and the ME3 had the higher values for vapor pressure deficit and the lower deficits by precipitation (**Figure 3D**).

**Figure 4:**
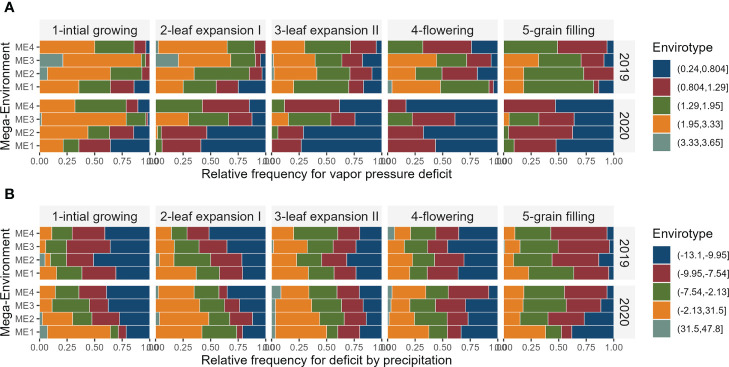
Relative frequency for each envirotype for vapor pressure deficit **(A)** and deficit by precipitation **(B)** observed in the studied and mega-environments across distinct crop stages and years of trials.

In ME1 during 2019, most parts of the days in the flowering and grain filling stages have vapor pressure deficit between 1.29 kPa d^-1^ and 3.33 kPa d^-1^. In this same ME in 2020, the vapor pressure deficit was smaller, with values ranging from 0.24 kPa d^−1^ and 0.804 kPa d^−1^ during ~50% of the days in the flowering and grain filling stages. Although 2019 presented on average lower precipitation (**Figure 3B**), the ME1 presented the higher deficit by precipitation, with positive values for almost ⅓ of the days during leaf expansion. In grain filling, for example, ~60% of the days had deficits that ranged from−7.54 mm day^−1^ to 31.5 mm day^−1^ (**Figure 4A**).

### 3.2 Variance components analysis

**Figure 5:**
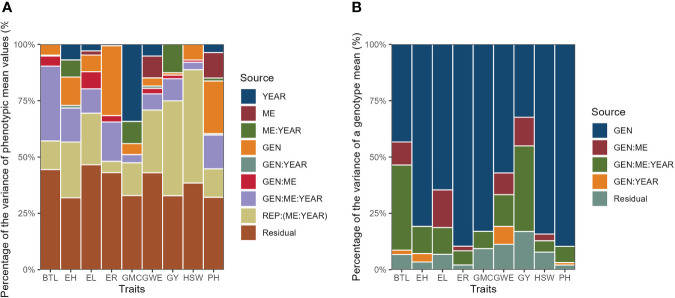
Percentage of the variance of phenotypic mean values **(A)** and percentage of the variance of a genotype mean **(B)**.

The likelihood ratio test of the deviance analysis revealed a significant (*P* ≤ 0.01) genotype effect for all the traits, except for GY and BTL (**Table 3**), suggesting good prospects of selection gains for most of the studied traits. The GEN × ME × YEAR interaction was significant for all the traits, with the highest contributions to the phenotypic variance of BTL, EH, ER, and PH (**Figure 5A**). The results suggests that those morphological traits are dependent on how the genotypes respond to different environmental stimuli. The ME × YEAR interaction was significant (*P* ≤ 0.01) for GMC and GY, suggesting that the contrasting climate variables observed across the two years affected the ME differently. Thus, it is reasonable to perform the selection within each ME. Overall, the REP (ME×YEAR) effect was significant for all the traits and was the component with the highest contribution for the phenotypic variance of GY. This high contribution likely comes from the implicit effect of location, since the location and complete blocks were combined to serve as replicates within each mega-environment. Here, although showing a high contribution, the location effect is not of primary interest, since the main goal is to identify superior genotypes within each mega-environment.

**Table 3:** Variance components for the main effect of genotypes ( ${\text{σ}}_{G}^{2})$ , mega-environments ( ${\text{σ}}_{M}^{2}$ ), cultivation year ( ${\text{σ}}_{Y}^{2}$ ), and their interactions estimated for nine traits assessed in 26 maize hybrids.

Source of variation	BTL^a^	EH	EL	ER	GMC	GWE	GY	HSW	PH
${\text{σ}}_{G}^{2}$	0.050^ns^	50.028**	0.211**	0.859**	1.357**	39.221*	0.038^ns^	1.770**	199.780**
${\text{σ}}_{M}^{2}$	0.000^ns^	0.000^ns^	0.053^ns^	0.000^ns^	0.000^ns^	104.484^ns^	0.000^ns^	0.000^ns^	96.522^ns^
${\text{σ}}_{Y}^{2}$	0.000^ns^	27.351^ns^	0.083^ns^	0.016^ns^	9.488*	55.866^ns^	0.000^ns^	0.000^ns^	31.061^ns^
${\text{σ}}_{GM}^{2}$	0.048^ns^	0.000^ns^	0.219**	0.078^ns^	0.000^ns^	26.481^ns^	0.059^ns^	0.250^ns^	0.000^ns^
${\text{σ}}_{GY}^{2}$	0.00 ^ns^	4.622^ns^	0.000^ns^	0.000^ns^	0.000^ns^	10.899^ns^	0.000^ns^	0.000^ns^	4.833^ns^
${\text{σ}}_{MY}^{2}$	0.000^ns^	30.248^ns^	0.000^ns^	0.000^ns^	2.738**	0.000^ns^	0.453*	0.000^ns^	11.210^ns^
${\text{σ}}_{GMY}^{2}$	0.353**	59.987**	0.311**	0.484**	1.011**	77.715**	0.355**	0.852**	128.617**
${\text{σ}}_{REP\left(M:Y\right)}^{2}$	0.135**	98.436**	0.657**	0.138**	4.033**	300.744**	1.521**	12.844**	108.140**
${\text{σ}}_{\epsilon }^{2}$	0.472	126.749	1.337	1.194	9.136	464.349	1.185	9.817	274.329
${\text{σ}}_{P}^{2}$	0.117	61.950	0.327	0.959	1.636	68.744	0.117	2.103	222.846
H_b_^2^	0.433	0.808	0.646	0.896	0.830	0.571	0.324	0.842	0.896
Ac	0.658	0.899	0.804	0.946	0.911	0.755	0.569	0.918	0.947

a BTL, bare tip length; EH, ear height; EL, ear length; ER, ear row; GMC, grain moisture content; GWE, grain weight per ear; GY, grain yield; HSW, 100-seed weight; and PH, plant height.

b Broad-sense heritability on the mean-basis.

^*^P ≤ 0.05; ^**^P ≤ 0.01 (See the P-values in **Supplementary Table S1**); ^ns^ P > 0.05.

The broad-sense heritability on the genotype-mean basis (H^2^) ranged from 0.324 (GY) to 0.896 (ER and PH) (**Table 3**). For the traits GY and BTL the H^2^ was less than 0.5, which means that the genotypic component accounted for less than 50% of the variance of a genotype mean (**Figure 5B**). For these traits, most of the variance of the genotype mean was due to both GEN × ME and GEN × ME × YEAR interactions. The greater contributions of interaction terms for these traits compared to the other ones reinforces that the phenotype-genotype relationship of this traits is strictly environment-dependent, which makes more difficult the selection of widely adapted hybrids and indicates that the selection within delineated mega-environments would provide better gains.

### 3.3 Correlation between traits in each mega-environment

**Supplementary Figure S7** shows the phenotypic correlations among the studied traits within each mega-environment over the two years. Overall, PH and EH were positively correlated with each other across all the MEs. Negative correlations were observed between PH and HSW, suggesting that taller plants have a lower density of grains. In ME1 and ME3 a negative correlation between GY and BTL (r = −0.13 and r = −0.12, respectively) was observed. For ME2 and ME3, a positive relation between GY and BTL was observed. These changes in the relationships in the different ME resulted in a low degree of Mantel’s correlation between the matrices (**Supplementary Figure S8**) (Guillot and Rousset, 2013). Therefore, this supports the use of a multi-trait index within each ME to take into account the different correlation structures.

### 3.4 Selection differentials for mean performance and stability

**Figure 6:**
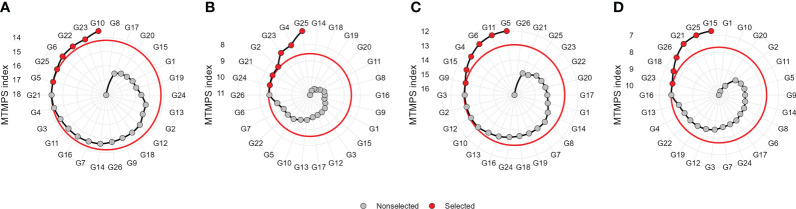
Genotype ranking and selected genotypes for the multi-trait mean performance and stability index (MTMPS) considering a selection intensity of 25% within ME1 **(A)**, ME2 **(B)**, ME3 **(C)**, and ME4 **(D)**. The red and black circles represent selected and unselected genotypes in their respective environments.

**Figure 7:**
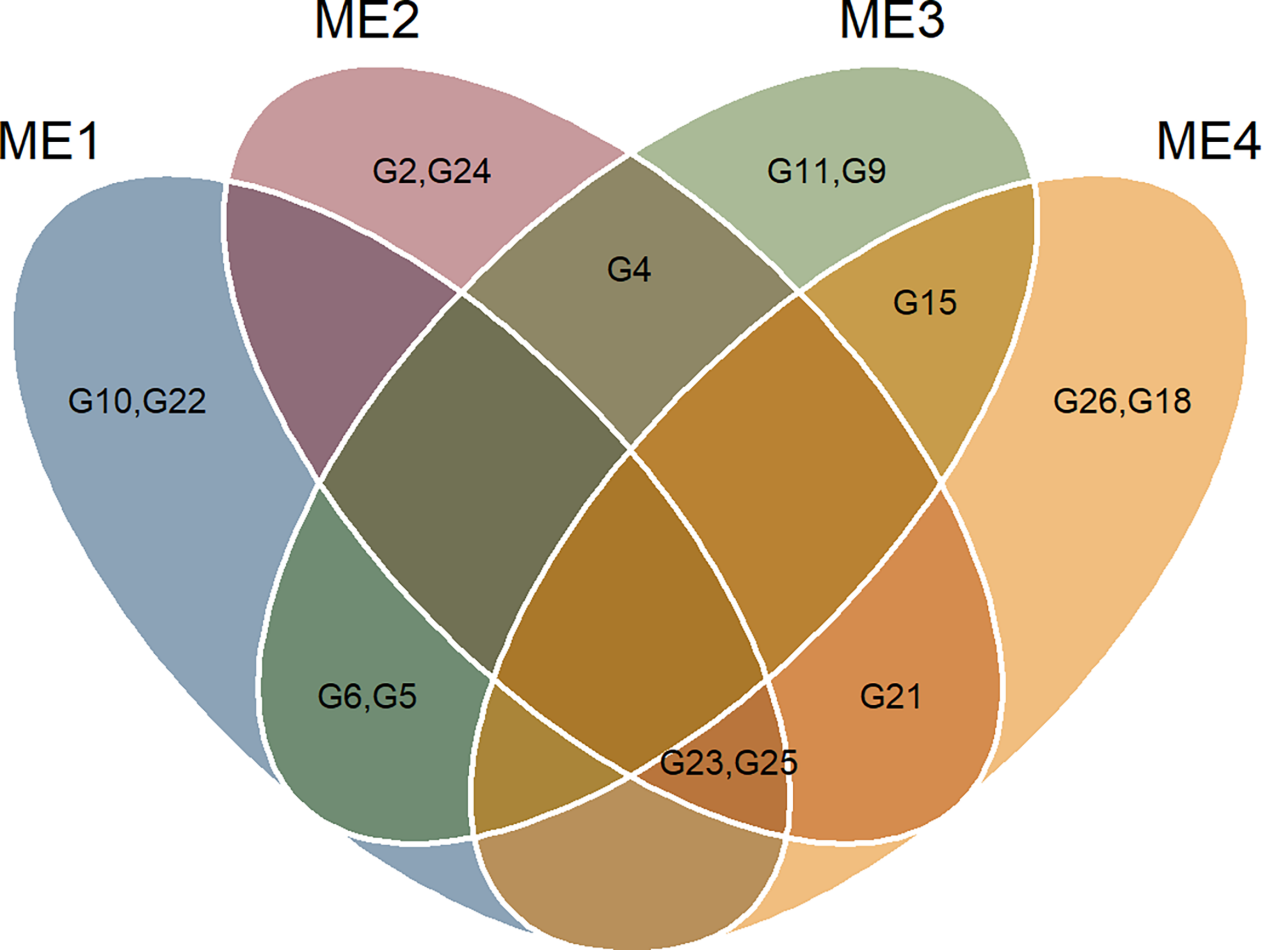
Veen plot showing the selected genotypes in each ME.

The selection considering the multiple traits resulted in different hybrids being selected in each ME (**Figures 6** and **7**). Overall, two hybrids were selected only for specific MEs, suggesting a narrow adaptation of such hybrids in such ME (**Figure 7**). Only two hybrids (namely, G23 and G25) were selected in three ME (ME1, ME2, and ME4). This suggests that these hybrids present a wide adaptation, performing well in different environments.

For all the MEs, four factors (FA) were retained, explaining 76.07%, 73.77%, 78.09%, and 73.64% of the total variance for ME1, ME2, ME3, and ME4, respectively (**Supplementary Table S2**). Given the different correlation structures (**Supplementary Figure S7**), different traits were included in each FA within each ME (**Supplementary Table S3**).

**Figure 8:**
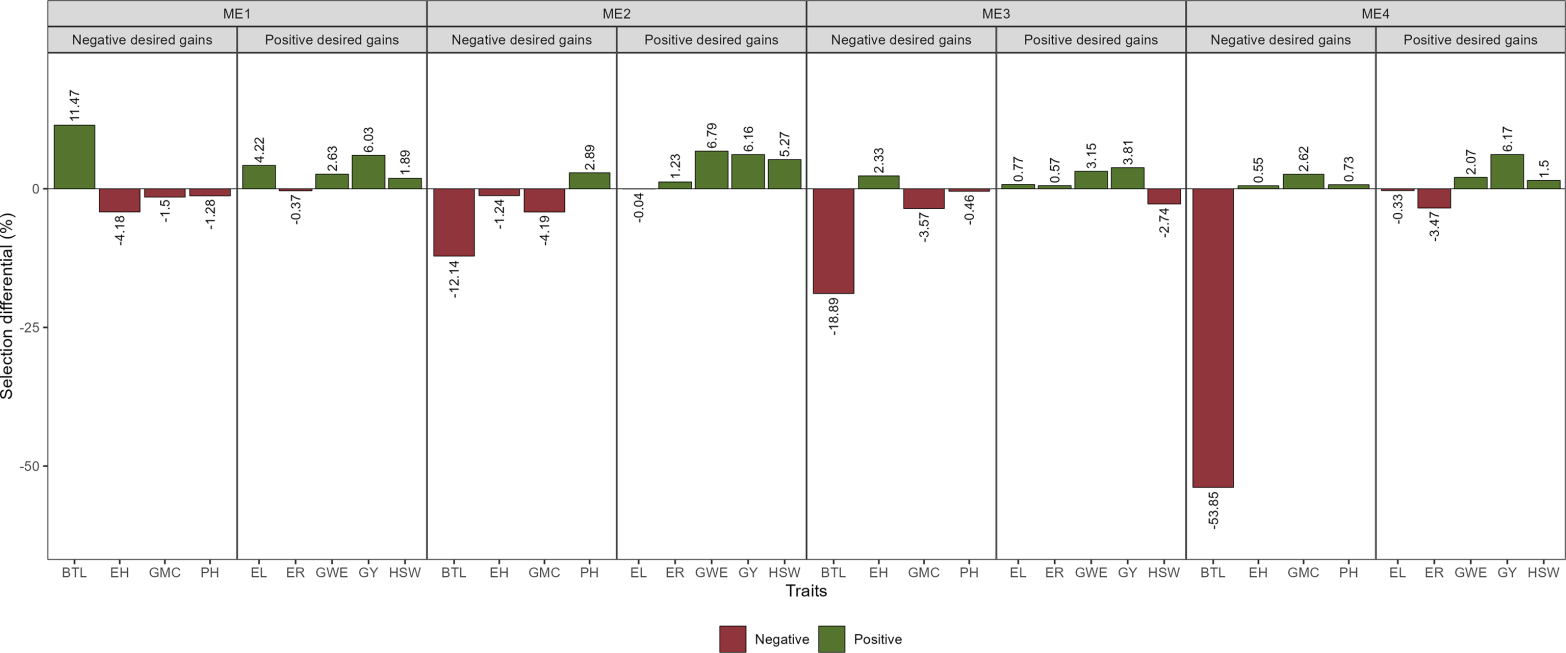
Selection gains for mean performance considering the selection within each ME.

The multi-trait selection resulted in a success rate in selecting traits with desired selection differentials (SD) of ~77% (7 out of 9 traits) in ME1, ME2, and ME3, and ~44% (4 out of 9 traits) in ME4 (**Figure 8**). The six selected maize hybrids (ranked by MTMPS) within ME1 were G10, G23, G22, G6, G25, and G5 (**Figure 6A**). In ME2, the selected hybrids were G25, G4, G23, G2, G21, and G26 (**Figure 6B**). In ME3 the selected hybrids were G5, G11, G6, G4, G15, and G9 (**Figure 6C**). For ME4, G15, G25, G21, G26, G18, and G23 were selected (**Figure 6D**).

**Figure 9:**
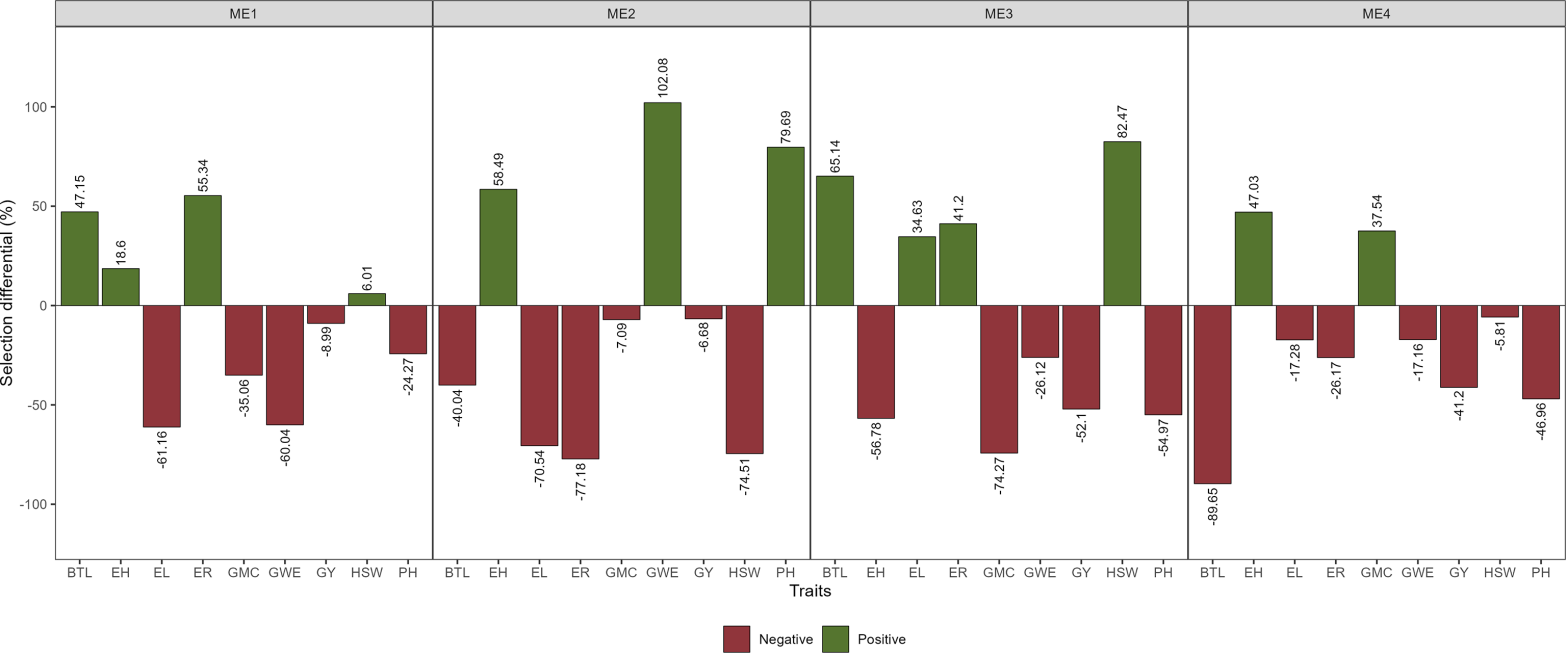
Selection gains for stability considering the selection within each ME.

The SD for BTL was negative for all ME except ME1. For GY positive SD that ranged from 3.81% in ME3 to 6.17% in ME4 were observed (**Figure 8**; **Supplementary Figures S9-12**; **Supplementary Table S3**). Considering the stability over the two cultivation years, negative SD was observed for most of the studied traits (**Figure 9**). For GY, negative SDs were observed in all the ME, with lower values for ME3 and ME4. These results show that the selected hybrids stand out as having satisfactory mean performance (average GY ranging from 10.38 Mg ha^-1^ in ME4 to 12.08 Mg ha-1 in ME1) with better stability across contrasting cultivation years.

### 3.5 Ranking the mega-environments

**Figure 10:**
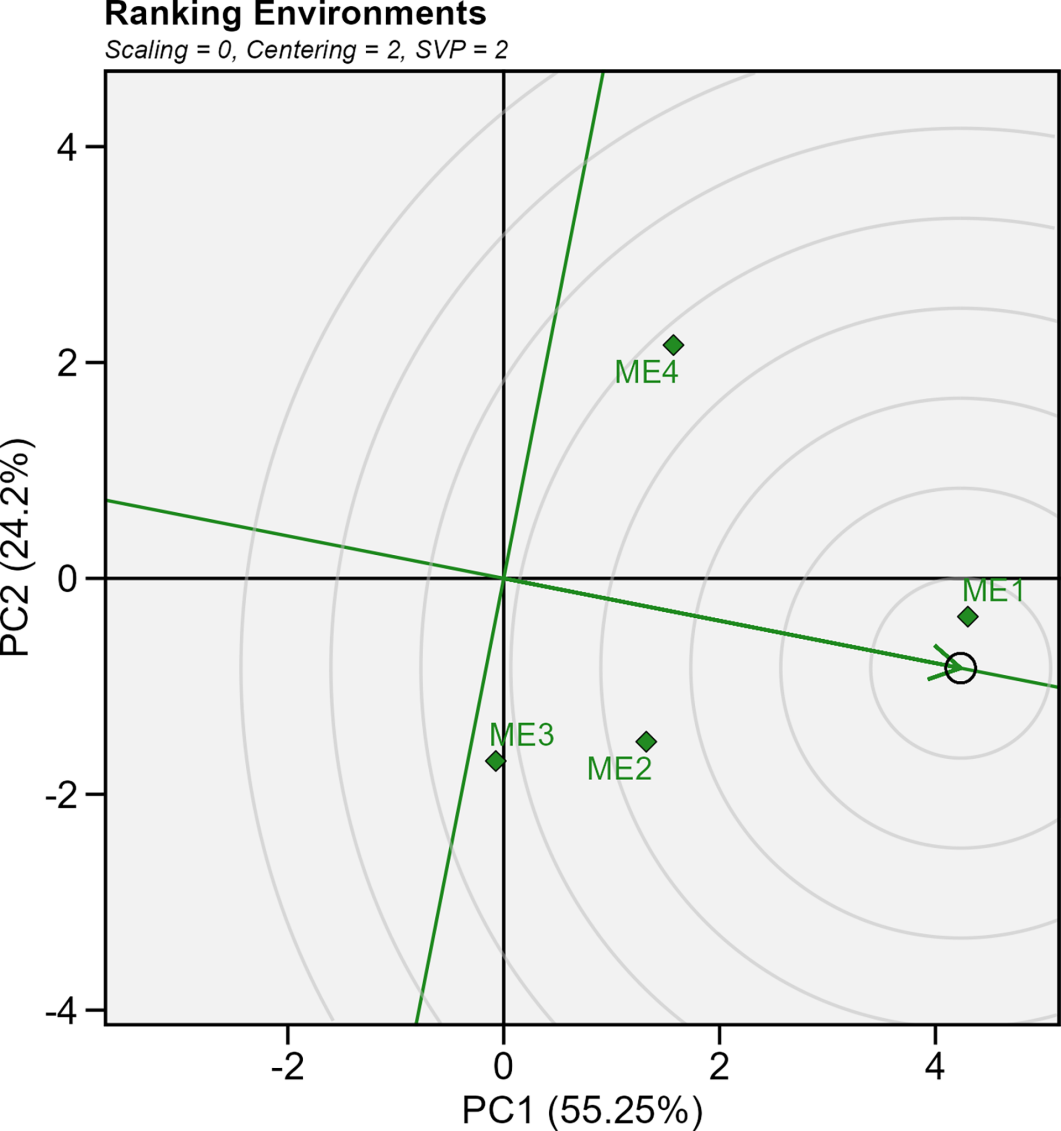
GGE biplot showing ranking of test mega-environment relative to an ideal test mega-environment.

**Figure 10** shows the genotype plus genotype-by-environment (GGE) biplot showing the ranking of the delineated mega-environments relative to an ideal mega-environment. Considering the average yield in each ME, the ME1 (which included only Yicheng) is closer to the “ideal” environment. On average, the yield in ME1 was 11.4 Mg ha^-1^ (**Supplementary Figures S13-14**). On the contrary, ME4 presented lower average yields (9.8 Mg ha^-1^), appearing far from the score of the “ideal” environment (**Figure 10**).

## 4 Discussion

### 4.1 Seasonal effects impacted the mega-environments differently

The 10 environments included in this study were categorized into 4 mega-environments (ME) by which the similarity was assessed on an “omics” scale of 19 environmental covariables with long-term (20 years) weather data (**Figure 2**). These results support previous studies that also identified the complex climatic conditions in HHH (Tao et al., 2017).

The two years of trials had contrasting climate characteristics (**Figures 3B, C**), which may be the source of the significant (*P*< 0.05) ME×YEAR interaction for GY (**Table 3**; **Supplementary Table S1**). In ME1, for example, a highly distinct pattern of vapor pressure deficit, minimum and maximum air temperatures across the crop cycle were observed in the two cultivation years. While in 2019 most of the flowering period in ME1 had a high vapor pressure deficit (**Figure 4A**) and maximum temperatures between 34.5°C and °C 39.3°C (**Supplementary Figure S15A**), 2020 had milder temperatures and a smaller vapor pressure deficit. This approach can leverage plant ecophysiology knowledge aiding to identify the main sources of the genotype-environment interaction to select stress-resilient hybrids (Costa-Neto and Fritsche-Neto, 2021; Resende et al., 2021; Carcedo et al., 2022).

In warm weather, the loss of water by evapotranspiration is greater than in colder weather. On average, ME1 experienced higher values of vapor pressure deficit, which by combining relative humidity and temperature into a single quantity (Penman and Keen, 1948) is an accurate measure for predicting plant transpiration (Seager et al., 2015). Surprisingly, ME1 2019 was the most productive environment with an average yield of 1.2 Mg ha^-1^ greater than the yield observed in 2020 at the same location (**Supplementary Figure 13**). The high yields in such environments lead to the hypothesis that the deficit by precipitation (**Figure 4B**), mainly during grain filling, was not sufficiently high to cause the limited transpiration rate trait, frequently expressed in some hybrids under high vapor pressure deficit and water-limited areas (Shekoofa et al., 2016). As a C4-metabolism plant, maize has a higher temperature optimum for photosynthesis than C3 plants due to the operation of a CO_2_-concentrating system that inhibits Rubisco oxygenase activity (Berry and Bjorkman, 2003). Previous studies have shown that maize net photosynthesis is only inhibited at leaf temperatures above 38°C and that the maximum quantum yield of photosystem II is relatively insensitive to leaf temperatures up to 45°C (Crafts-Brandner and Salvucci, 2002). When leaf temperature is increased gradually, rubisco activation and net photosynthesis acclimate by the expression of a new activase polypeptide. This acclimation may have occurred in ME1 since maximum air temperatures > 32°C were observed for most of the days since leaf expansion and may explain why this environment presented a high yield even with ~75% of the flowering period experiencing temperatures > 34.5 °C (**Supplementary Figure S15A**).

Another climate variable that can explain the higher yield of ME1 in 2019 is the minimum temperatures. In 2020, ~75% of the days during the grain filling stage had minimum air temperatures below 19.2°C (**Supplementary Figure S15B**), which resulted in a negative correlation between GY and minimum temperature (**Figure 3C**). Previous studies have shown that temperatures below 20°C rose abruptly the redox state of the primary electron acceptor of photosystem II (QA), and increase the non-photochemical quenching of chlorophyll fluorescence, suggesting a restriction in electron transport in such conditions (Labate et al., 1990; Sowiński et al., 2020).

### 4.2 Higher precipitation does not ensure higher yields

Overall, the environments in 2020 presented accumulated rainfall during the experiment greater than 500 mm, researching ~920 mm in ME1 (**Supplementary Figure S16**). As a result, these environments showed a higher deficit by precipitation (positive deficits mean more water availability), and a lower vapor pressure deficit (**Figure 4B**). Unexpectedly, higher yields were not observed in such environments. In Jieshou, for example, the average yield was 2.6 t ha^-1^ smaller in 2020 compared to 2019, even with rainfall precipitation ~2.4-fold higher, with 387 mm in 2019, and 917 mm in 2020 (**Supplementary Figure S16**).

A possible explanation for the lower yields observed in environments with higher water availability may be related to the restricted plant transpiration in such cases. Water moves from the soil into plant roots, and by negative pressures within the xylem due to capillary forces in the cell walls, to the leaves. The water, warmed by the sun, turns into vapor passing out through stomata, at the same time that allows absorption of CO_2_ to photosynthesize (Taiz and Zeiger, 2010). The propulsive force of this process is regulated by the difference in the concentration of water vapor between the intercellular spaces of the leaves and the external atmospheric mass; the energy of this process is provided by the amount of radiation available. In ME1 during 2020, for example, ~87% of the grain filling period presented relative humidity greater than 70% (**Supplementary Figure S15C**). In addition, the wind speed in such a location had the lowest average (0.20 m s^-1^). The combination of high relative humidity and lower wind speed might have dramatically reduced plant transpiration. While limitation on transpiration at high vapor pressure deficit is a promising trait that could be incorporated into breeding programs to improve drought tolerance in maize (Yang et al., 2012), lower yield under elevated air relative humidity may be related to the hindered acquisition of mineral nutrients, mainly those supplied to plant roots by mass flow (NO_3_^−^, Ca^+2^, and Mg^+2^), considering the transpiration-driven mass flow concept (Cramer et al., 2009).

### 4.3 Envirotyping helped to better understand the genotype-environment interaction

The significant GEN×ME×YEAR interaction suggests the complex interaction of the genotypes with contrasting environments on the trait phenotypic expression. Similar reports were also observed in previous studies (Kamutando et al., 2013; Mebratu et al., 2019; Yue et al., 2020; Singamsetti et al., 2021). Along with the global changes in climatic variables over the past decades, there is a growing consensus that future food production will be threatened by environmental conditions (Ceglar and Kajfež-Bogataj, 2012; Steward et al., 2018). Therefore, knowledge about the influence of climatic variables on maize yield and genotypic variation within a certain environment is particularly necessary. Among the climatic factors investigated, temperature, vapor pressure deficit, deficit by precipitation, and relative humidity were key environmental factors to distinguish yield across different environments, which in turn affects GE interactions (de Araujo et al., 2019).

Growing resilient crops with consistent yield performance in unpredictable and complex weather changes is critical to ensuring food security. Given the large scale of production, high degree of mechanization, and developed biotechnology, coupled with measures and technical means such as reasonably dense planting, scientific fertilization, biological pest control, and water-saving irrigation, the maize yield level of Unites States of America has long been among the highest in the world (~10.5 Mg ha^-1^). China’s maize yield in 2020 was 6.35 Mg ha^-1^ which was 60% of the US yield level (Guo et al., 2021). Given the huge difference in corn production between China and the United States, how to select and breeding excellent corn hybrids that adapt to the climate characteristics of different ecological regions is the key to ensuring the healthy and stable development of China’s corn industry (Yue et al., 2022b). Since the 1960s, the Dryland Farming Institute, Hebei Academy of Agriculture and Forestry Sciences has been focusing on the breeding of new high-yield and stable maize hybrids and the multi-environment trials of summer maize in the HHH Plain, making full use of foreign germplasm resources to improve local germplasm. A series of excellent summer maize hybrids were selected and bred.

Here, we provided evidence that using envirotyping techniques to define mega-environments based on climate variables may help breeders to better understand the genotype-by-environment interaction. Several studies define mega-environments based on the genotypes’ response in a single year (Singh et al., 2019; Mushayi et al., 2020; Enyew et al., 2021), but since the environmental pattern that defines the genotype response may change significantly across years (**Figure 4**), this may lead to mistaken recommendations. In most studies that evaluate genotypes across multiple locations and years, the average yield across years is used to fill a two-way table (genotypes x locations) that is further used in AMMI or GGE biplot analysis (Shojaei et al., 2022). Here, we have shown how integrating multi-trait selection for mean performance (within mega-environments) and stability (across years) with detailed environmental typology may be useful to identify specific adaptations (such as tolerance to warmer environments), increasing the sustainability of breed programs mainly under the climate changes in view (Lopes et al., 2015). Therefore, our results can leverage plant ecophysiology knowledge aiding in identifying the primary sources of the genotype-environment interaction in plant breeding programs (Costa-Neto and Fritsche-Neto, 2021; Resende et al., 2021). The use of this approach becomes particularly interesting due to the dynamism and exhaustivity of the data available (climate information available for all points of the globe) that make it possible to replicate the procedure anywhere, anytime, and the possibility of including additional information such as soil proprieties, crop management, companion organisms, and crop canopy (Xu, 2016).

### 4.4 The multi-trait selection provided desired gains for most of the studied traits

To the best of our knowledge, this is the first introduction of the term multi-trait mean performance and stability index (MTMPS). The MTMPS can be seen as an adaptation of the MTSI (Olivoto et al., 2019b) where several parametric and non-parametric stability measures (beyond the WAASB) can be used. Similar to the MTSI, genotypes that have lower values of MTMPS are assumed to have better mean performance and stability based on the set of accessed traits. Multi-trait stability index has recently been employed as a robust tool to assist the selection of elite genotypes based on the mean performance and stability of various variables. Some examples include the selection of resistant soybean genotypes to drought and salinity (Zuffo et al., 2020), bread wheat ideotypes for adaptation to early sown conditions (Farhad et al., 2022), barnyard millet lines for shoot fly resistance (Padmaja et al., 2022), drought tolerant chickpea genotypes (Hussain et al., 2021), pea lines adapted to autumn sowings in broomrape-prone Mediterranean environments (Rubiales et al., 2021), and maize inbred lines under optimal and drought stress conditions (Balbaa et al., 2022).

A key factor in using the MTMPS is choosing an adequate *θ*_*Y*_/*θ*_*s*_ ratio for each trait, which will likely change the genotype ranking. By plotting the genotype ranks in several scenarios of *θ*_*Y*_/*θ*_*s*_ ratio (**Supplementary Figure S17**) it is possible to identify groups of genotypes with similar performance regarding stability and productivity. For example, in ME1, G10 and G23 (selected by the MTMPS) remained well-ranked regardless of the *θ*_*Y*_/*θ*_*s*_ ratio. This suggests that they have both high yield and satisfactory stability. On the other hand, G8 remained poorly ranked either considering only the mean performance or stability (**Supplementary Figure S17**). The poor performance for GY –and possibly for all the other traits– made this genotype the last ranked within ME1 (**Figure 6A**). In our case, highly stable hybrids across years could be identified as those that are better ranked when *θ*_*Y*_/*θ*_*s*_ tends to 0. One example in ME1 would be G21, which was the top-ranked when only stability was considered in the MPS (**Supplementary Figure S17**).

Here, we found that the use of the MTMPS provided desired gains for the mean performance and stability for most of the studied traits (**Figures 8** and **9**) and that the selection within mega-environments with similar climatic patterns may provide satisfactory gains. The use of MTMPS is expected to grow rapidly among breeders helping to identify hybrids that combine desired mean performance and stability for important traits. For example, envirotyping and morpho-physiological and yield traits accessed [eg., Balbaa et al. (2022)] can be combined to identify stress-adaptive traits with a high yield and helps to better understand the genotype-by-environment interaction.

## 5 Conclusions

Considering 20 years of climate information and 19 environmental covariables, we identified four mega-environments (ME) for maize cultivation in the Huanghuaihai plain in China. Overall, most of the studied traits were significantly affected by genotype × mega-environment × year interaction. The vapor pressure deficit, maximum temperature, relative humidity, and deficit by precipitation were the climate variables that most contributed to the envirotyping. This provides relevant evidence that evaluating maize stability and adaptation to mega-environments using single-year, multi-environment trials may provide misleading recommendations. Counterintuitively, higher yields were not observed in the environments with higher accumulated rainfall precipitation. We provide strong pieces of evidence that vapor pressure deficit, minimum temperatures, and relative humidity may be climate variables that –in environments with no water restriction–, have a relevant control on the plant transpiration and consequently, yield. Utilizing the MTMPS approach in this study led to identifying six different selected hybrids in each mega-environment, with higher stability and prominent mean performance for most of the studied traits. G23 and G25 were selected within three out of the four mega-environments, being identified as stable. The G5 shows satisfactory yield and stability across contrasting years in the drier, warmer, and with higher vapor pressure deficit mega-environment, which included locations in the Hubei province. To the best of our knowledge, this is the first study that integrated envirotyping techniques and multi-trait selection for mean performance and stability, opening the door to a more systematic and dynamic characterization of the environment to better understand the genotype-by-environment interaction in multi-environment trials.

## Data availability statement

The source code used to produce the static website and the results in this article have been archived at https://doi.org/10.5281/zenodo.7221255 as manuscript_v3.0.0 (Olivoto, 2022).

## Author contributions

HY: Conceptualization, data curation, formal analysis, funding acquisition, investigation, methodology, software, visualization, writing-original draft, writing-review & editing. TO: Conceptualization, statistical analysis, writing- review & editing. JB: Conceptualization, data curation, formal analysis. JL: Data curation, formal analysis. JW: Conceptualization, writing original draft, writing-review & editing. JX: Conceptualization, data curation, formal analysis, funding acquisition, methodology, supervision. SC: Conceptualization, data curation, formal analysis, funding acquisition, methodology, supervision, validation. HP: Conceptualization, data curation, funding acquisition, methodology, supervision. MN: Conceptualization, statistical analysis. XJ: Conceptualization, data curation, formal analysis, funding acquisition, investigation, methodology, software, visualization, writing-original draft, writing-review & editing. All authors contributed to the article and approved the submitted version.

## Funding

This study was supported by the Key Research and Development Projects of Hebei Province (20326305D), Special Fund for National System (Maize) of Modern Industrial Technology (CARS-02), the Science and Technology Support Program of Hebei Province (16226323D-X), HAAFS Science and Technology Innovation Special Project, “Three-Three-Three Talent Project” Funded Project in Hebei Province (A202101056), the Agricultural Science and Technology Achievement Transformation Project of Hebei province (21626310D), the National Key Research and Development Program of China (2019YFE0120400) and Natural Science Foundation of Shandong Province, China (ZR2021MC107).

## Acknowledgments

The authors are grateful to the K. M. Chen (Assistant Professor, Meteorological Bureau of Hengshui, Hebei, China) for compiling and collecting meteorological data of this research.

## Conflict of interest

The authors declare that the research was conducted in the absence of any commercial or financial relationships that could be construed as a potential conflict of interest.

## Publisher’s note

All claims expressed in this article are solely those of the authors and do not necessarily represent those of their affiliated organizations, or those of the publisher, the editors and the reviewers. Any product that may be evaluated in this article, or claim that may be made by its manufacturer, is not guaranteed or endorsed by the publisher.
