# Supplementary material for: Multi-trait selection for mean performance and stability of maize hybrids in mega-environments delineated using envirotyping techniques
Source: Front Plant Sci. 2022 Nov 14;13:1030521. doi: 10.3389/fpls.2022.1030521 (PMC9702090; doi:10.3389/fpls.2022.1030521)
Supplement: Supplementary file 1 [file DataSheet_1.pdf]

Supplementary Material

1 Supplementary Figures

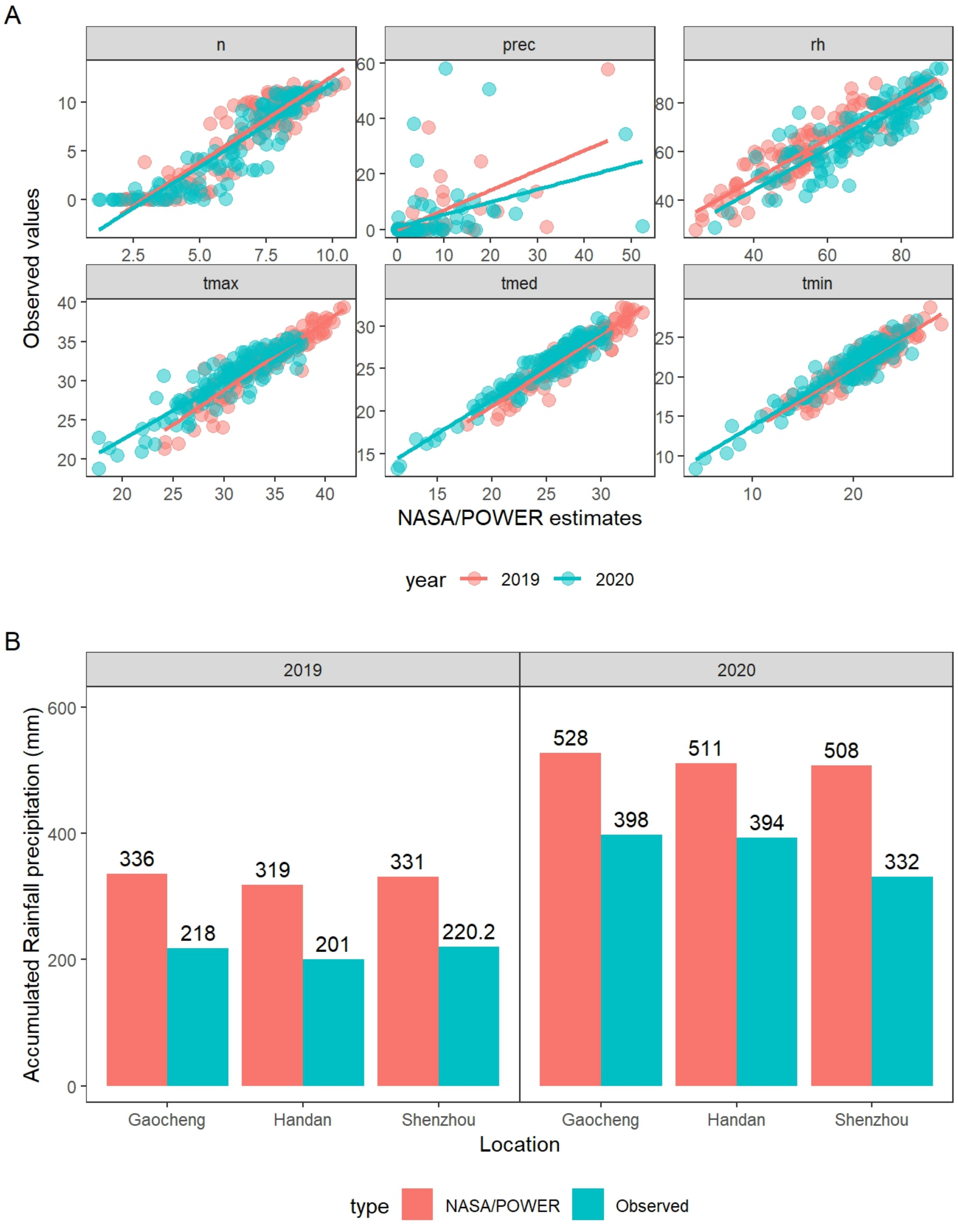

Supplementary Figure S1. NASA/POWER estimates vs observed values. (A) Relationship between NASA/POWER estimates and observed values (observed in Shenzhou) for daylight hours (n), rainfall precipitation (prec), relative humidity (rh), maximum air temperature (tmax), average air temperature (tmed), and minimum air temperature (tmin); (B) NASA/POWER vs observed values for accumulated rainfall in three different locations.

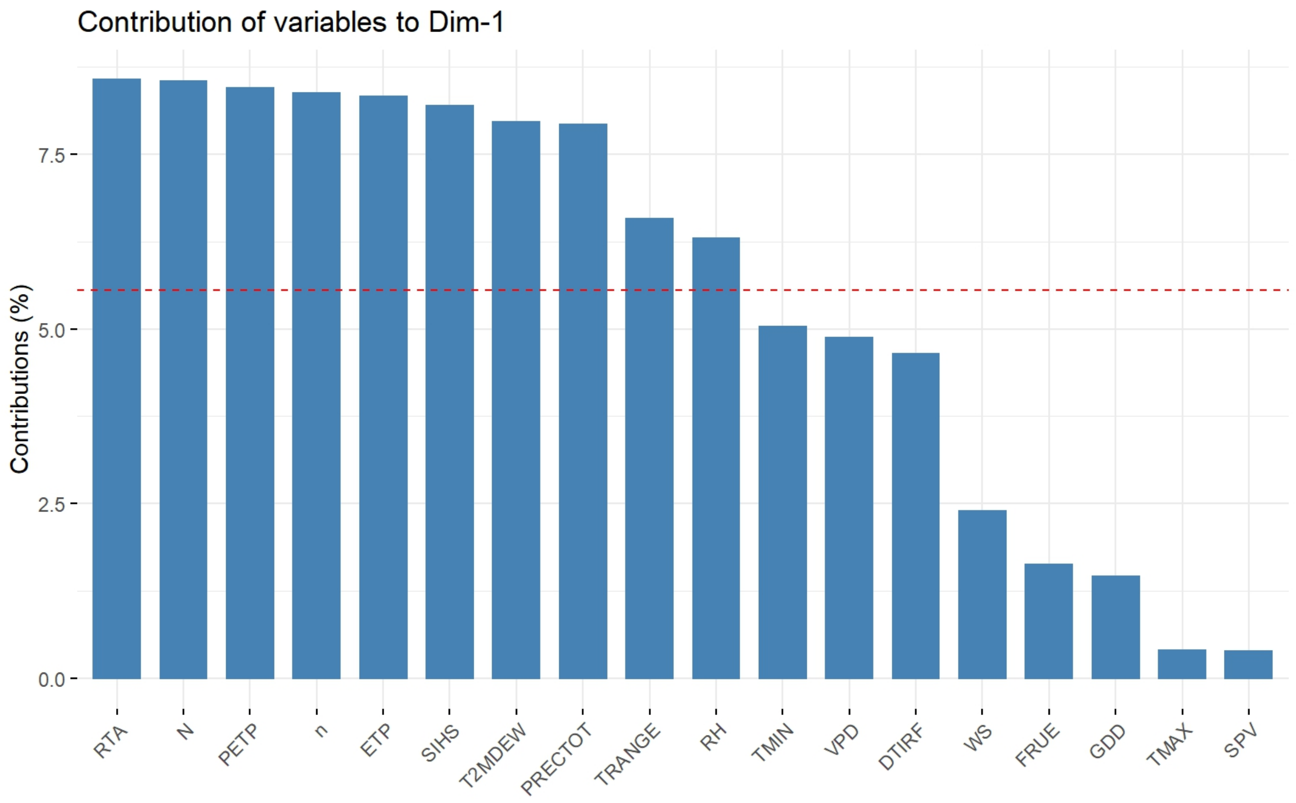

Supplementary Figure S2. The contribution of variables in the Principal Component Analysis (average values of 20 years).

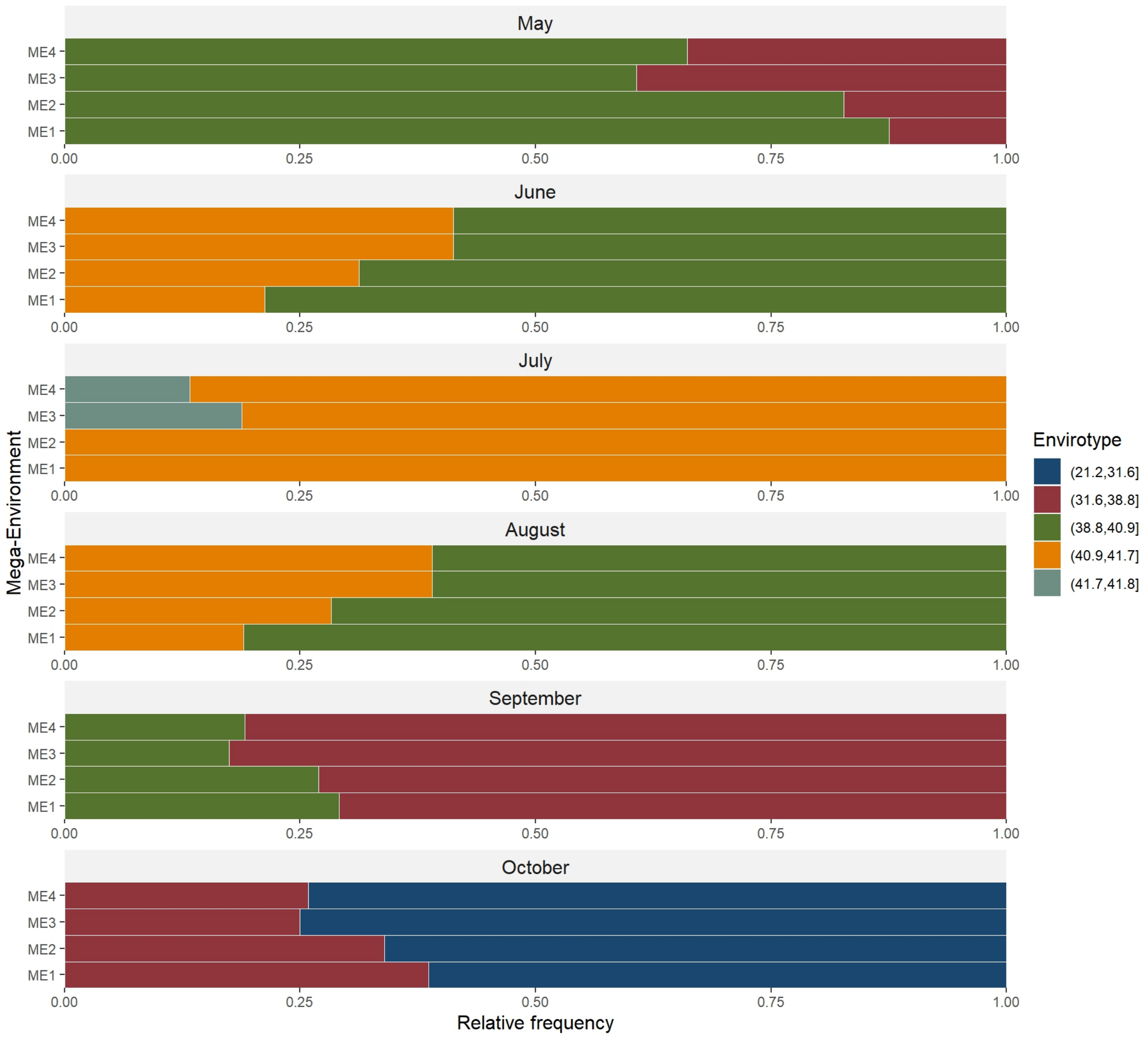

Supplementary Figure S3. Quantiles for extraterrestrial radiation over six months observed in the delineated mega-environments.

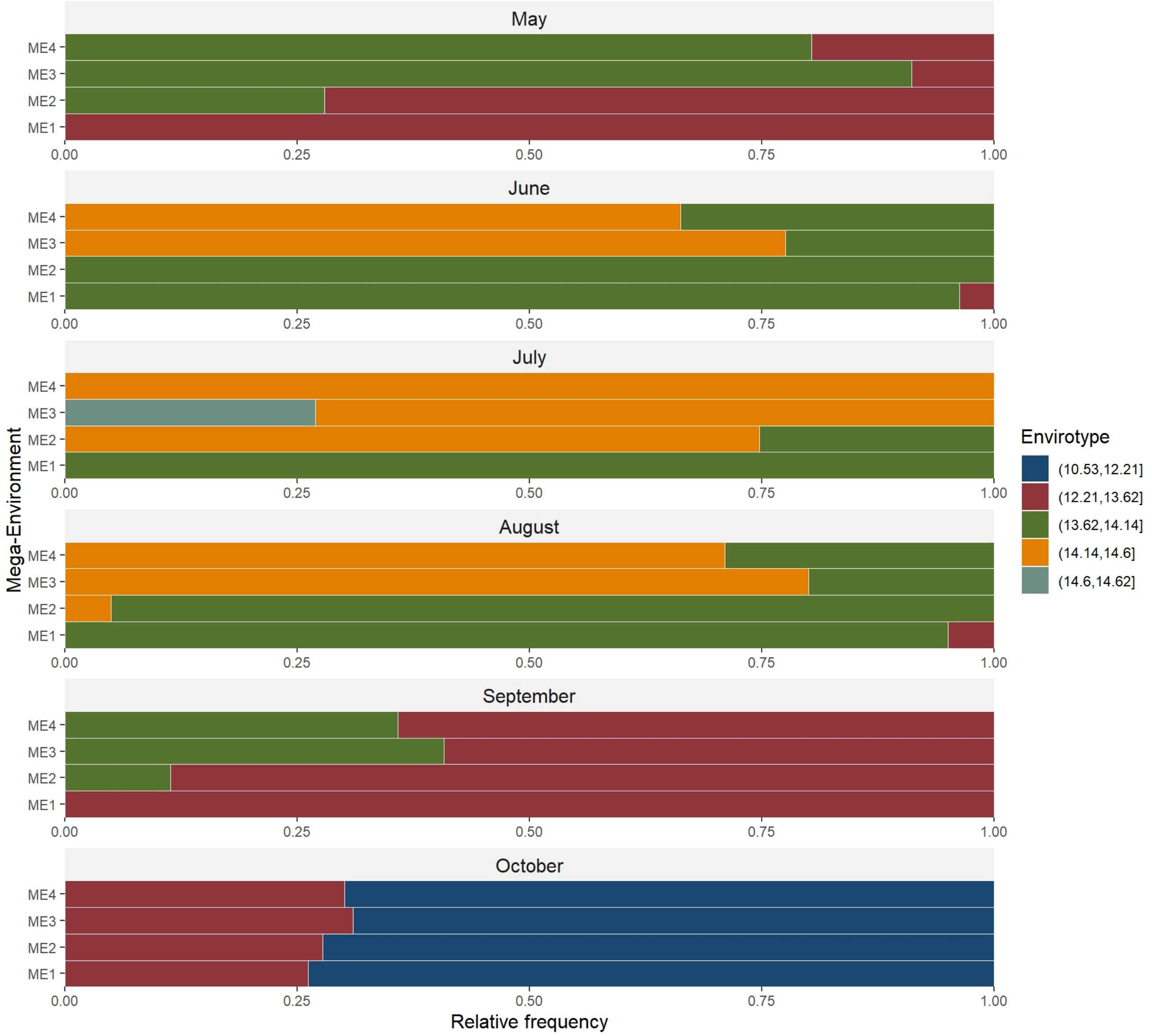

Supplementary Figure S4. Quantiles for daylight hours over six months observed in the delineated mega-environments.

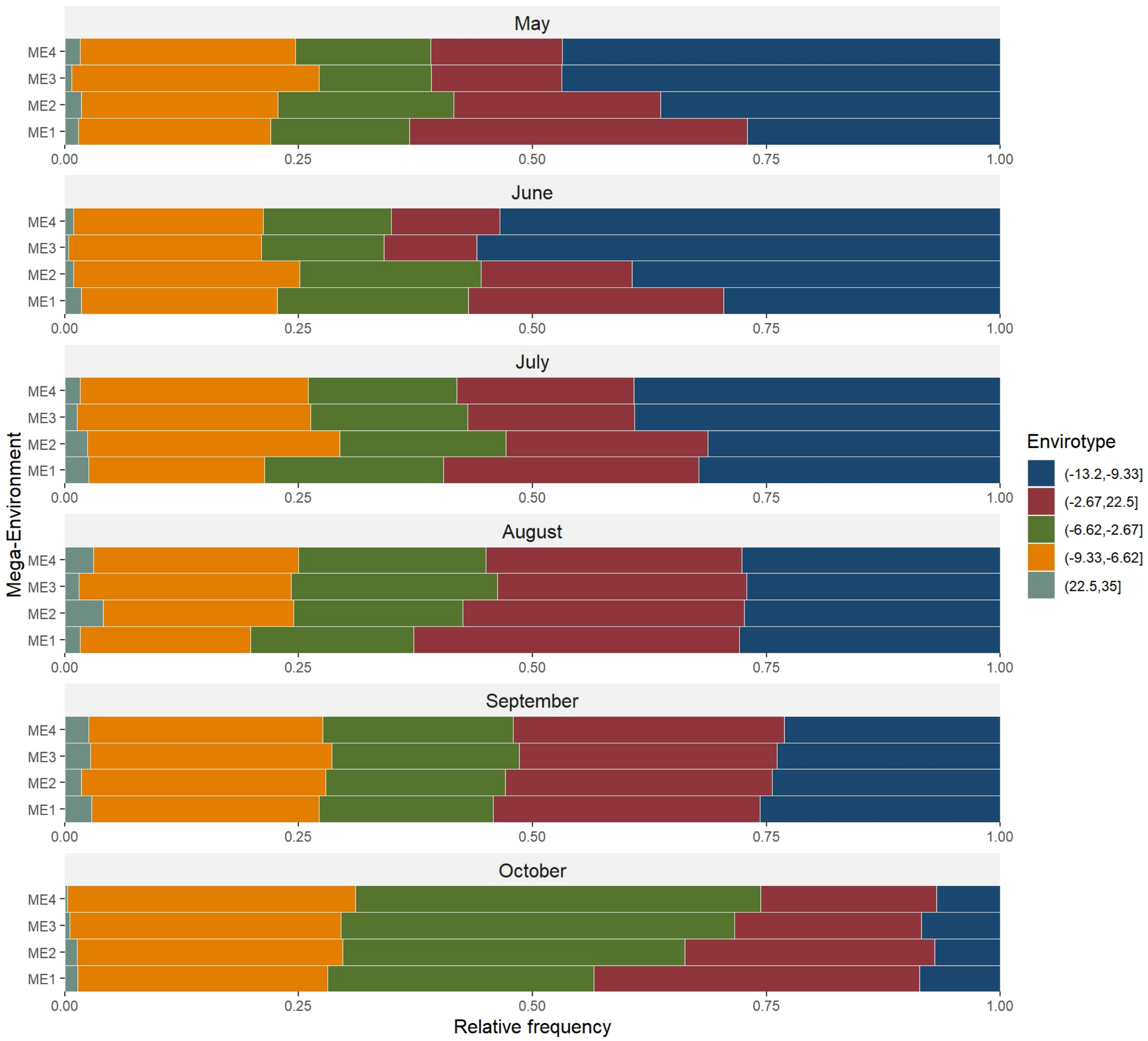

Supplementary Figure S5. Quantiles for deficit by precipitation over six months observed in the delineated mega-environments.

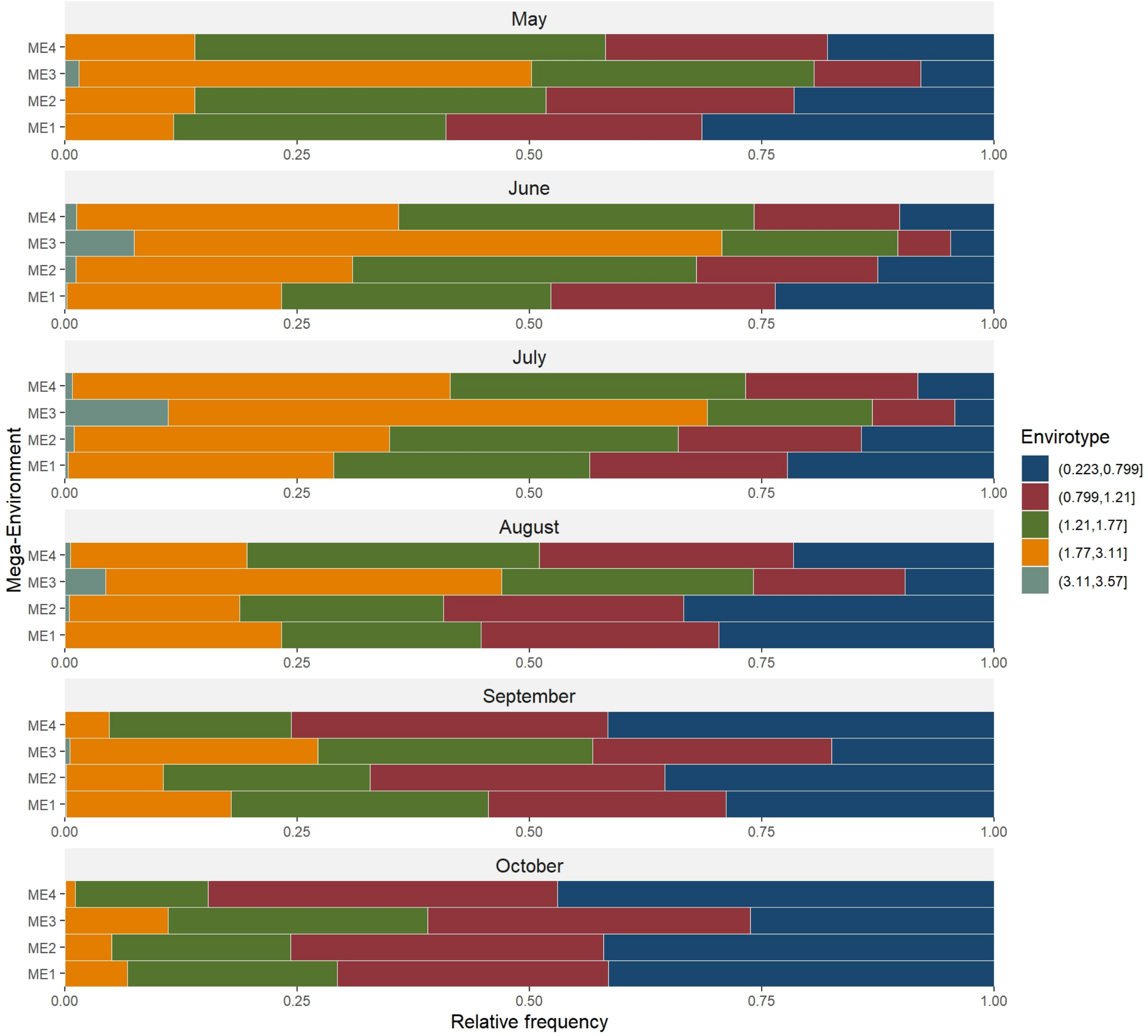

Supplementary Figure S6. Quantiles for vapor pressure deficit over six months observed in the delineated mega-environments.

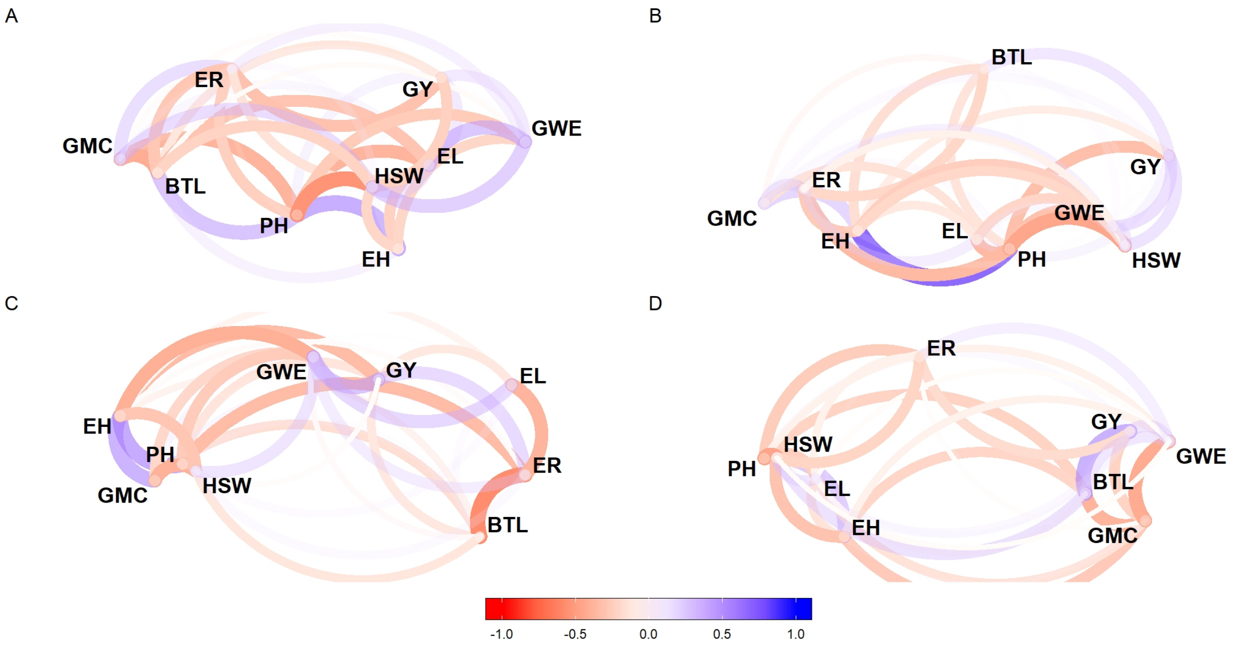

Supplementary Figure S7. Phenotypic correlation between the studied traits within ME1 (A), ME2 (B), and ME3 (C), and ME4 (D).

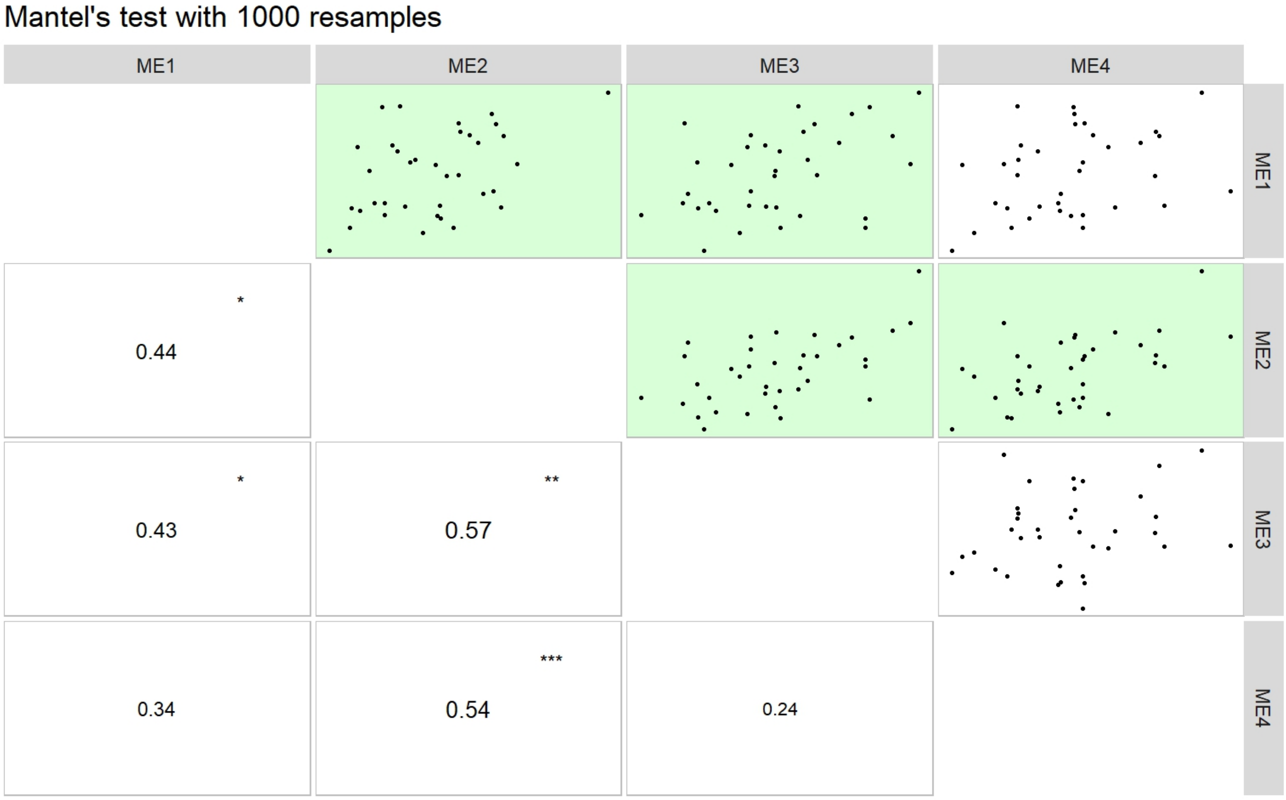

Supplementary Figure S8. Mantel's correlation between the phenotypic correlation matrices observed in each mega-environment.

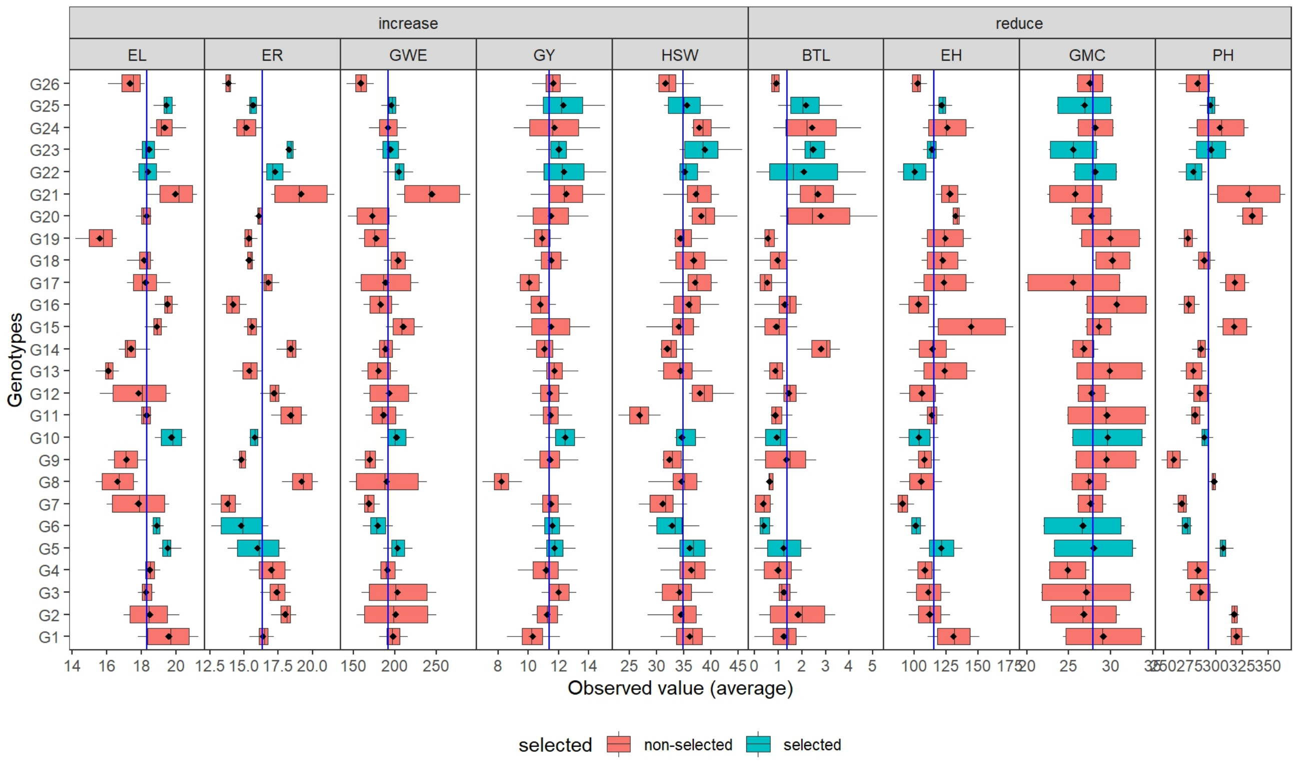

Supplementary Figure S9. Average value for the studied trait across ME1.

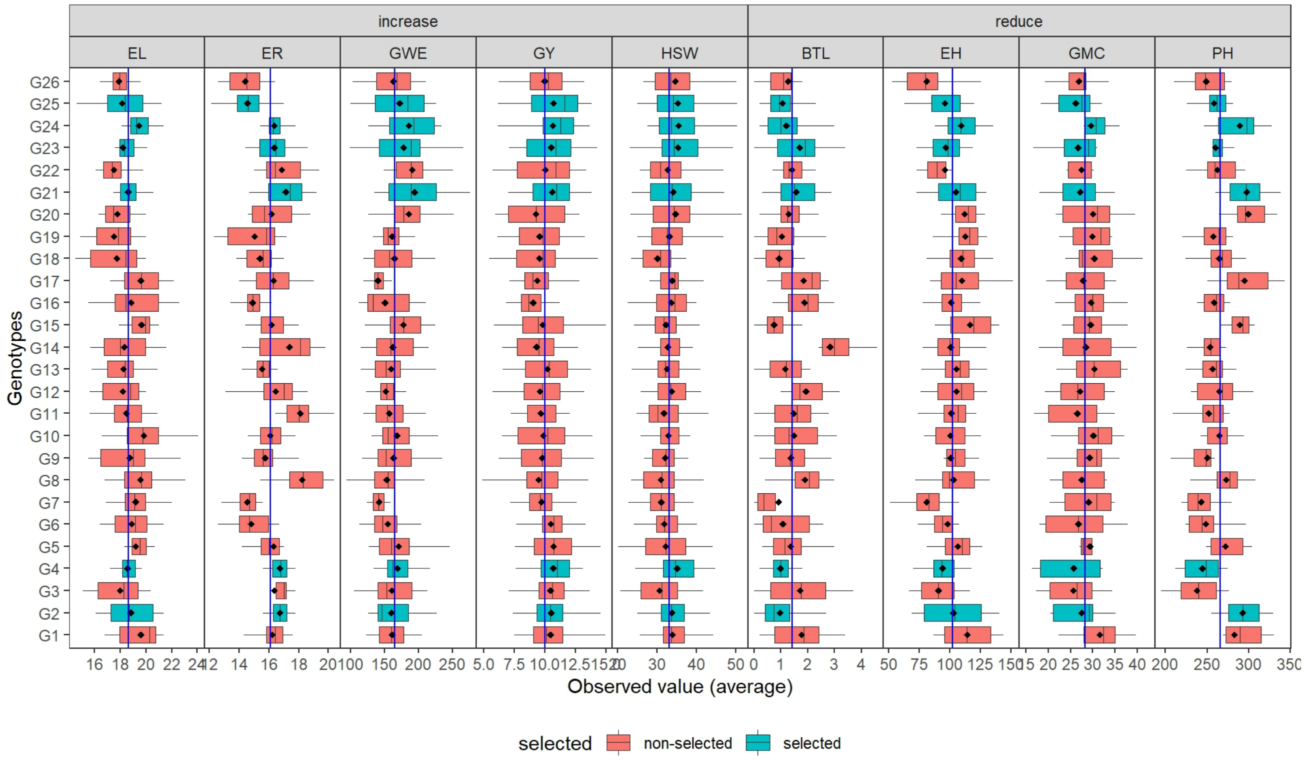

Supplementary Figure S10. Average value for the studied trait across ME2.

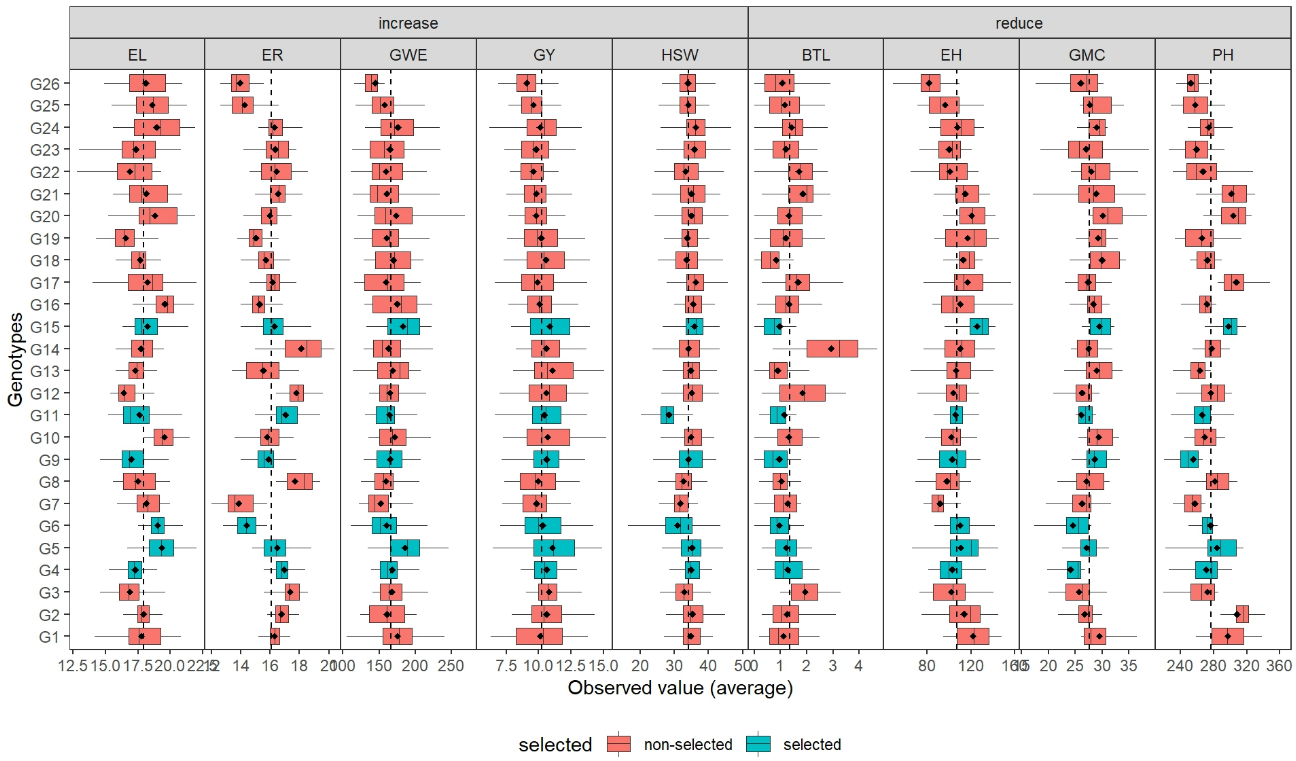

Supplementary Figure S11. Average value for the studied trait across ME3.

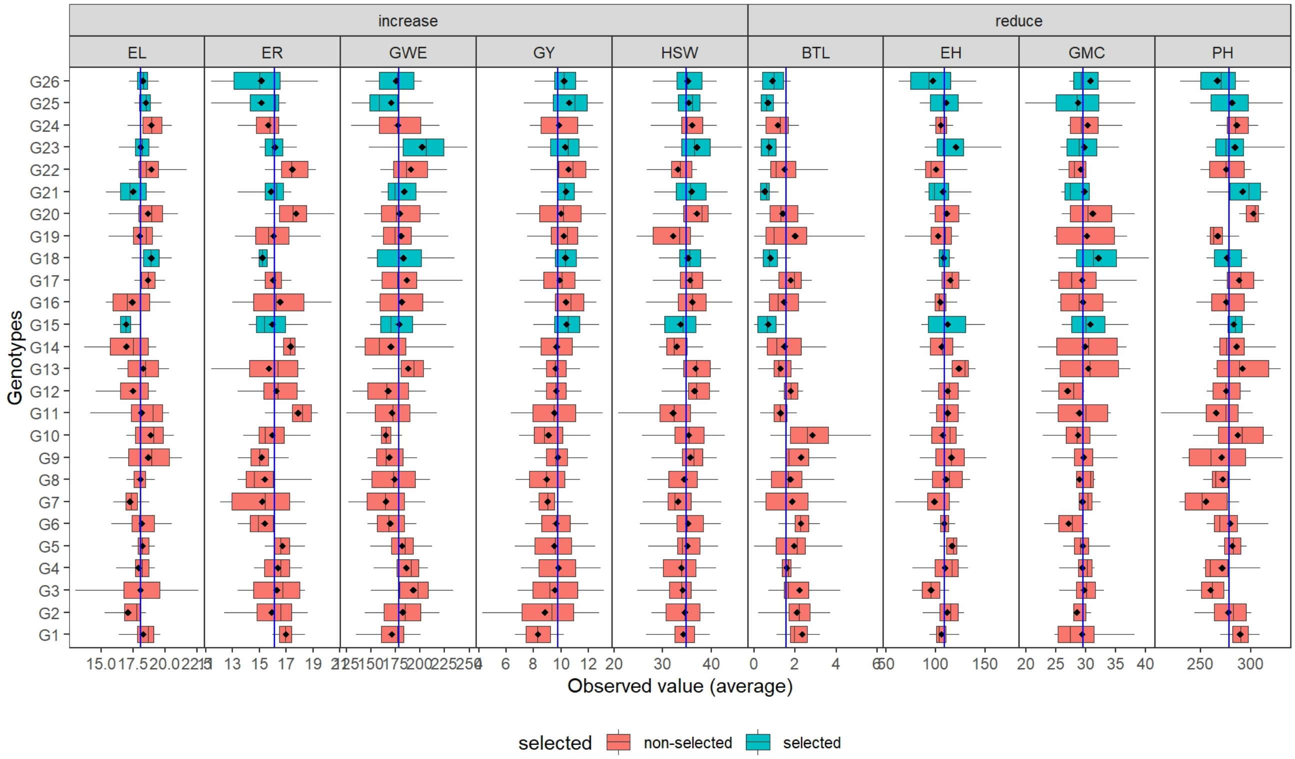

Supplementary Figure S12. Average value for the studied trait across ME4.

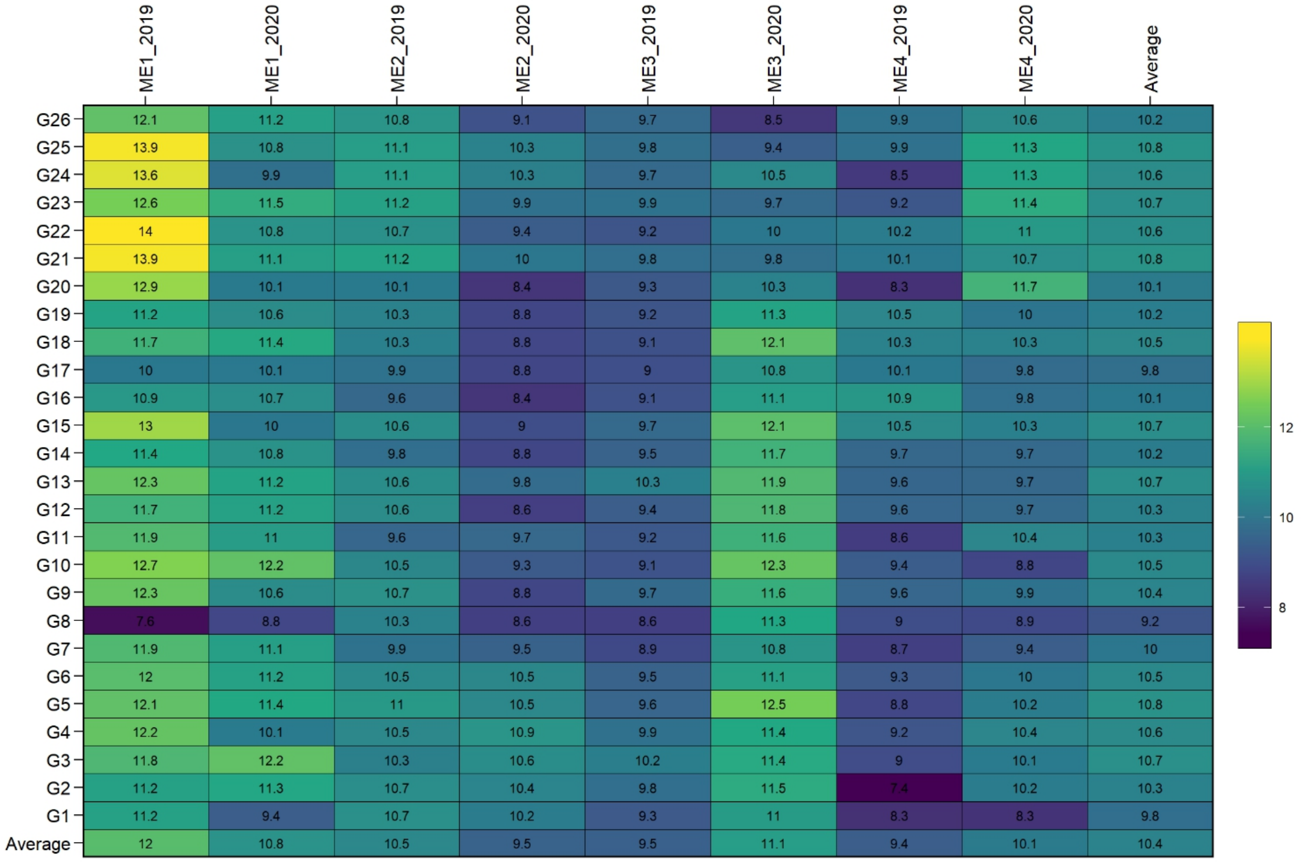

Supplementary Figure S13. Heatmap showing the average yield of the studied genotypes in each combination of mega-environment and year.

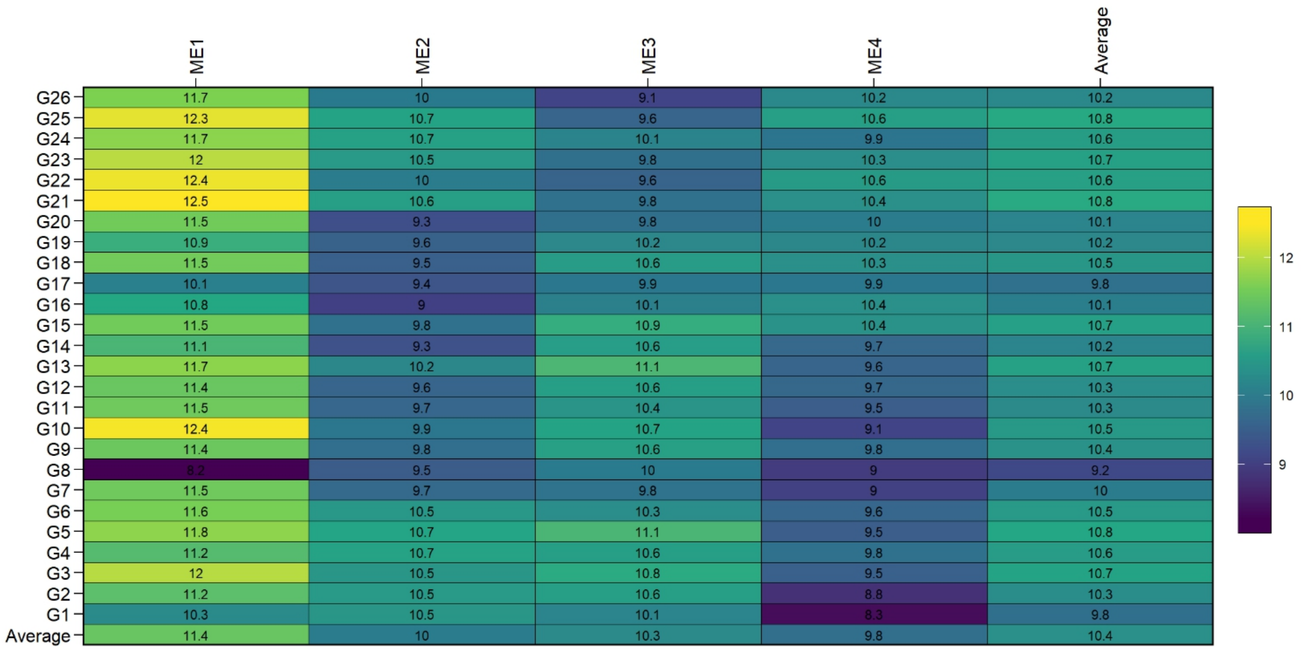

Supplementary Figure S14. Heatmap showing the average yield of the studied genotypes in each mega-environment.

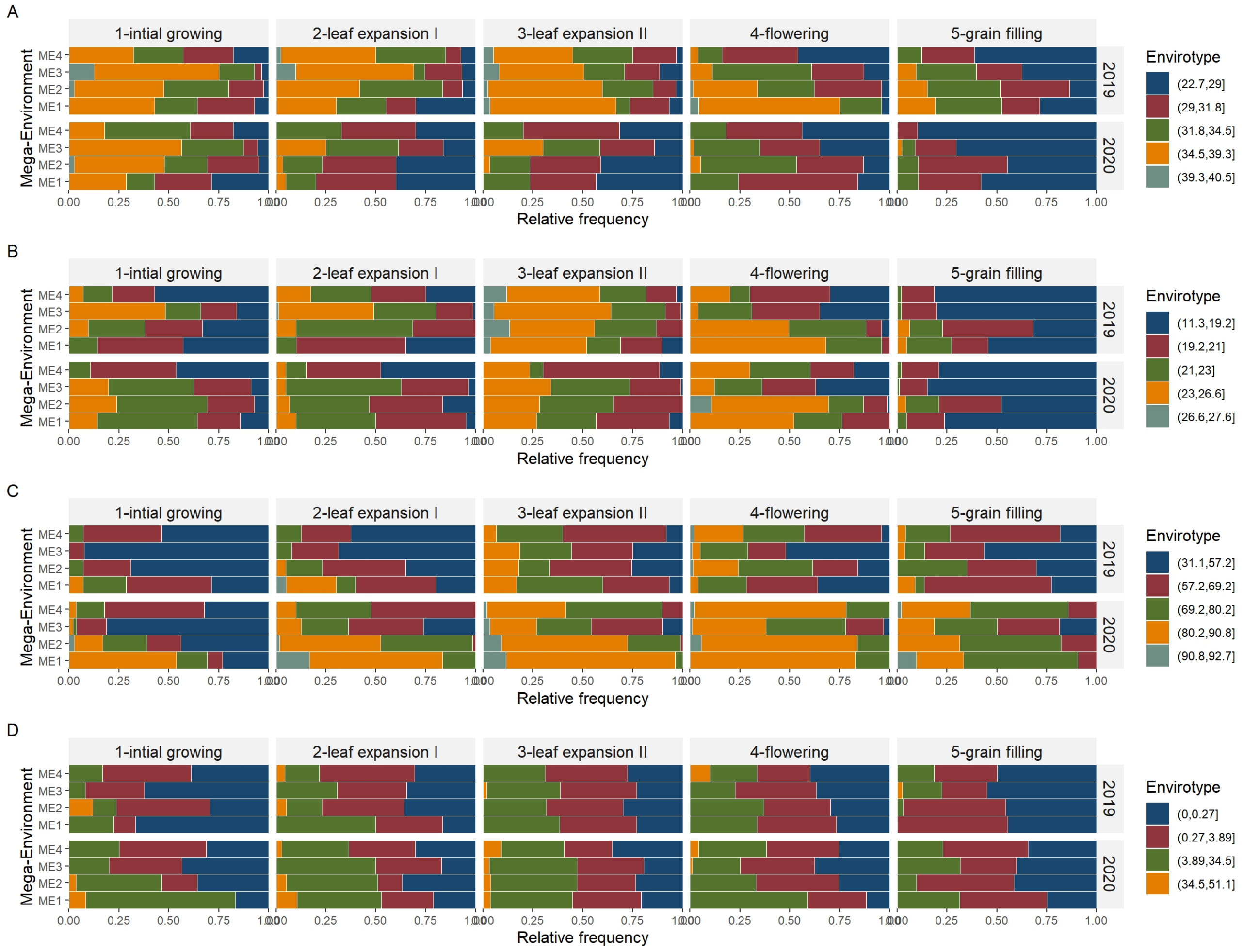

Supplementary Figure S15. Quantiles for maximum air temperature (A), minimum air temperature (B), relative humidity (C), and rainfall precipitation (D) observed in the studied mega-environments across distinct crop stages and cultivation years.

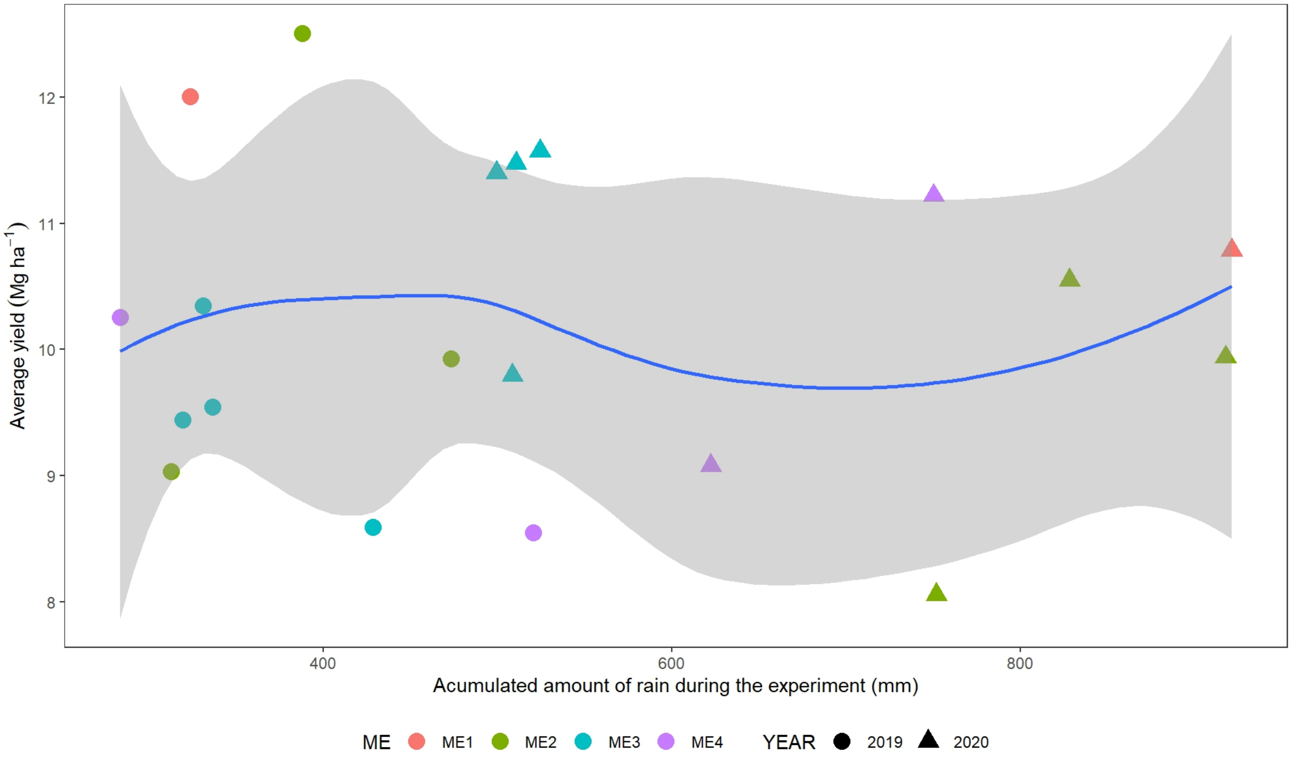

Supplementary Figure S16. Relationships between accumulated rainfall precipitation and grain yield. The different colors shows the mega-environments and the different shapes shows the cultivation year.

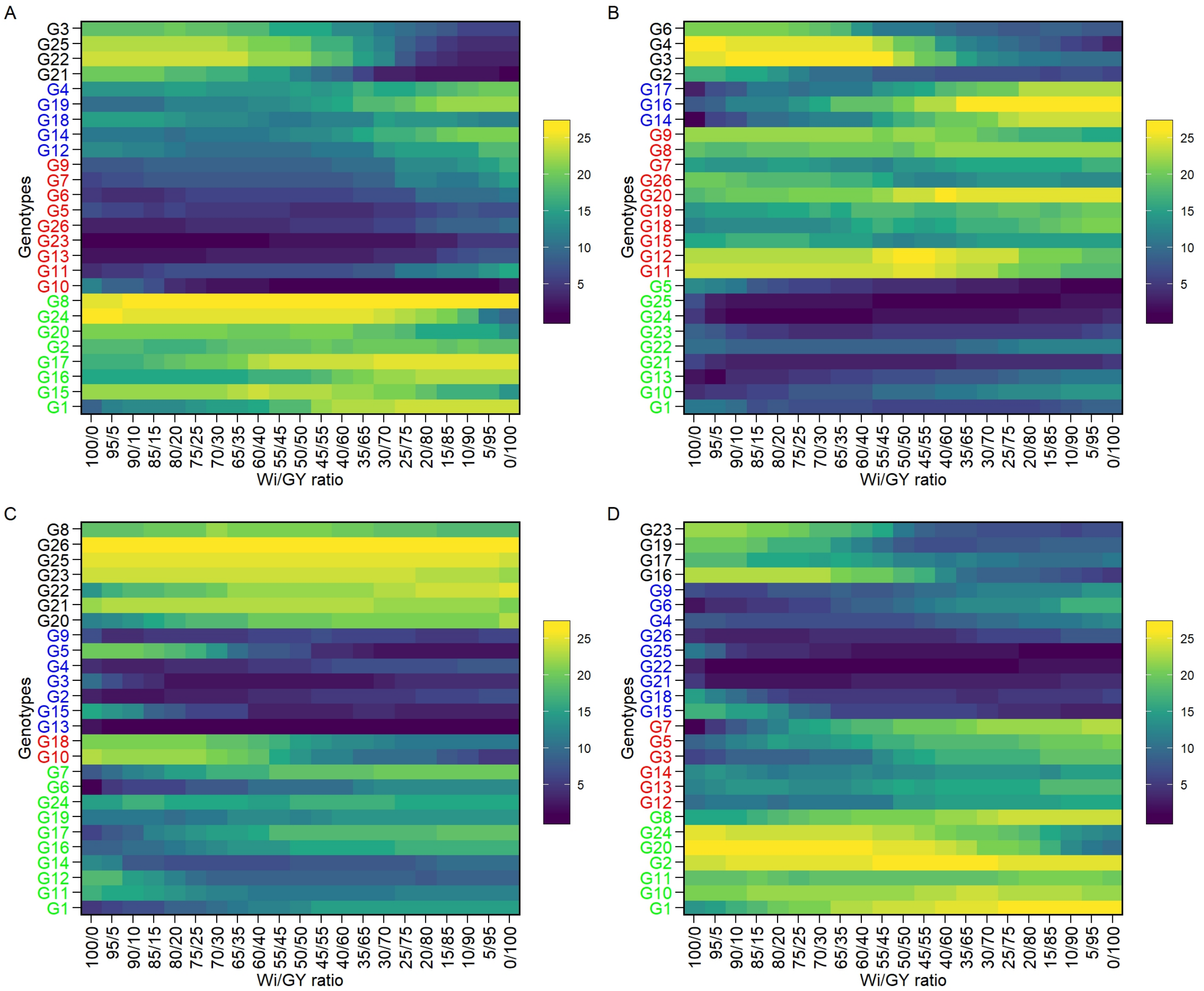

Supplementary Figure S17. Ranks of 26 maize hybrids considering different weights for stability computed as the Wricke's Ecovalence (Wi) and grain yielding (GY). The most-left ranks were obtained considering the stability only. The most right-ranks were obtained considering the grain yield only. Between the extremes, the ranks were obtained different weights for stability and yielding.

2 Supplementary Tables

Supplementary Table 1. Likelihood Ratio Test (LRT) for the random effects for each analyzed trait.

Source of variation	BTL	EH	EL	ER	GMC	GWE	GY	HSW	PH
ME	0.9993	0.998	0.4532	0.9999	1	0.1762	0.9996	1	0.1295
GEN	0.1623	0.000116	0.001976	2.81E-08	2.38E-05	0.02818	0.3131	1.23E-07	3.09E-07
GEN:YEAR	0.8979	0.486	1	1	1	0.4008	0.9999	0.9988	0.7332
GEN:ME	0.3916	1	0.005561	0.3466	1	0.1961	0.3527	0.3534	1
GEN:ME:YEAR	6.02E-38	5.85E-36	1.62E-11	1.41E-21	2.25E-06	4.09E-07	2.60E-17	0.000116	3.11E-32
REP:(ME:YEAR)	2.40E-54	6.921e-136	2.86E-91	3.49E-19	3.10E-81	3.42e-115	7.102e-204	2.899e-220	2.97E-72
ME:YEAR	1	0.09063	1	1	0.000667	1	0.0317	1	0.5888
YEAR	1	0.3594	0.2755	0.4651	0.04146	0.1256	0.9992	0.9993	0.2946

Supplementary Table 2. Eigenvalues and explained variance for the factor analysis within each mega-environment (ME1, ME2, ME3, and ME4).

Model	PC	Eigenvalues	Variance (%)	Cum. variance (%)
ME1	PC1	2.9048	32.2755	32.2755
ME1	PC2	1.659	18.4335	50.709
ME1	PC3	1.2536	13.9292	64.6381
ME1	PC4	1.0292	11.435	76.0732
ME1	PC5	0.8384	9.3154	85.3886
ME1	PC6	0.5137	5.7075	91.0961
ME1	PC7	0.4369	4.8547	95.9508
ME1	PC8	0.2353	2.6143	98.5652
ME1	PC9	0.1291	1.4348	100
ME2	PC1	2.6264	29.1824	29.1824
ME2	PC2	1.4635	16.2612	45.4436
ME2	PC3	1.3912	15.4574	60.901
ME2	PC4	1.158	12.8671	73.768
ME2	PC5	0.9121	10.1344	83.9024
ME2	PC6	0.6959	7.7324	91.6348
ME2	PC7	0.439	4.8783	96.5131
ME2	PC8	0.2292	2.5472	99.0603
ME2	PC9	0.0846	0.9397	100
ME3	PC1	2.6731	29.7011	29.7011
ME3	PC2	1.9628	21.8091	51.5102
ME3	PC3	1.312	14.5781	66.0883
ME3	PC4	1.0805	12.0054	78.0937
ME3	PC5	0.6778	7.531	85.6247
ME3	PC6	0.5212	5.7916	91.4162
ME3	PC7	0.4507	5.0078	96.4241
ME3	PC8	0.1951	2.1682	98.5923
ME3	PC9	0.1267	1.4077	100
ME4	PC1	2.2929	25.4767	25.4767
ME4	PC2	1.8789	20.8771	46.3538
ME4	PC3	1.3836	15.3734	61.7272
ME4	PC4	1.0722	11.9138	73.6411
ME4	PC5	0.7253	8.0584	81.6994
ME4	PC6	0.6302	7.0026	88.702
ME4	PC7	0.5051	5.6123	94.3144
ME4	PC8	0.3232	3.5909	97.9052
ME4	PC9	0.1885	2.0948	100

Supplementary Table 3. Selection differentials for mean performance and stability of the studied traits in the four delineated mega-environments.

			Mean performance			Stability		
**ME**	**TRAIT**	**Factor**	**Xo**	**Xs**	**SD**	**Xo**	**Xs**	**SD**
ME1	EH	FA1	115.169	110.358	-4.1767	287.915	341.479	18.6042
ME1	PH	FA1	292.901	289.167	-1.275	315.307	238.79	-24.267
ME1	EL	FA2	18.3032	19.075	4.2167	3.1804	1.2354	-61.156
ME1	GY	FA2	11.3963	12.0839	6.0336	2.278	2.0733	-8.9884
ME1	BTL	FA3	1.3955	1.5556	11.4684	3.1218	4.5937	47.1494
ME1	ER	FA3	16.3603	16.3	-0.3683	3.4559	5.3685	55.3442
ME1	GMC	FA3	27.9077	27.4889	-1.5007	9.5114	6.1766	-35.061
ME1	GWE	FA4	191.661	196.708	2.6335	1484.62	593.274	-60.039
ME1	HSW	FA4	34.8949	35.5528	1.8854	12.9523	13.7309	6.0113
ME2	EH	FA1	102.194	100.926	-1.2413	1746.17	2767.48	58.4883
ME2	GMC	FA1	28.3406	27.1537	-4.188	18.3819	17.0779	-7.094
ME2	PH	FA1	266.066	273.759	2.8914	3237.58	5817.54	79.6879
ME2	GY	FA2	10.002	10.6186	6.1646	2.0422	1.9058	-6.6775
ME2	HSW	FA2	33.0491	34.7907	5.2697	24.0236	6.1234	-74.511
ME2	BTL	FA3	1.4226	1.25	-12.136	2.5058	1.5026	-40.036
ME2	ER	FA3	16.1157	16.3139	1.2296	5.166	1.1787	-77.184
ME2	EL	FA4	18.6455	18.6389	-0.0355	1.3509	0.3979	-70.544
ME2	GWE	FA4	165.572	176.811	6.7879	959.933	1939.83	102.079
ME3	EH	FA1	107.394	109.896	2.3294	174.679	75.4959	-56.78
ME3	GMC	FA1	27.7042	26.7139	-3.5745	13.0603	3.361	-74.266
ME3	HSW	FA1	34.1939	33.2556	-2.7442	15.2821	27.8854	82.4718
ME3	PH	FA1	276.478	275.215	-0.4566	1433.67	645.597	-54.969
ME3	BTL	FA2	1.3433	1.0896	-18.886	1.3436	2.2187	65.1352
ME3	ER	FA2	16.1045	16.1958	0.5672	3.0241	4.2699	41.198
ME3	EL	FA3	17.9563	18.0951	0.7735	4.7299	6.3681	34.6328
ME3	GWE	FA4	166.067	171.297	3.1494	994.12	734.483	-26.117
ME3	GY	FA4	10.2719	10.6635	3.8122	6.9039	3.3072	-52.098
ME4	BTL	FA1	1.5679	0.7236	-53.85	3.043	0.3149	-89.651
ME4	GMC	FA1	29.5715	30.3458	2.6186	32.3595	44.5085	37.5439
ME4	GWE	FA1	178.691	182.383	2.0665	863.388	715.242	-17.159
ME4	GY	FA1	9.7761	10.3792	6.1689	3.6834	2.1659	-41.199
ME4	EH	FA2	108.942	109.542	0.5502	291.328	428.335	47.0283
ME4	HSW	FA2	34.8843	35.4083	1.5022	10.2273	9.6333	-5.808
ME4	PH	FA2	278.833	280.875	0.7322	700.043	371.334	-46.956
ME4	ER	FA3	16.1497	15.5889	-3.4725	5.7913	4.2754	-26.175
ME4	EL	FA4	18.0981	18.0389	-0.327	4.5881	3.7955	-17.276

ME, mega-environment; FA, factor retained; Xo, mean of the original population; Xs, mean of the selected genotypes; SD, selection differential; GY, grain yield; GMC, grain moisture content; PH, plant height; EH, ear height; EL, ear length; ER, ear row; BTL, bare tip length; GWE, grain weight per ear and HSW, 100-seed weight.
